# Integrated Genomic and Transcriptomic Profiling of Isolated Trisomies in AML Reveals Cell Cycle Dysregulation and Therapeutic Vulnerabilities

**DOI:** 10.1111/jcmm.70941

**Published:** 2025-11-27

**Authors:** Jersey Heitor da S. Maués, Bruno Kosa L. Duarte, Maria Carolina C. M. Svidnicki, Herton Luiz A. S. Filho, Fernanda Soares Niemann, Adriana da Silva S. Duarte, Paula de Melo Campos, Pedro M. Moraes‐Vieira, Sara Teresinha Olalla Saad

**Affiliations:** ^1^ National Institute of Science and Technology of Blood (INCT) and Hematology and Transfusion Medicine Center (Hemocentro) University of Campinas Campinas Brazil; ^2^ Laboratory of Immunometabolism, Department of Genetics, Evolution, Microbiology and Immunology–Institute of Biology University of Campinas (UNICAMP) Campinas Brazil

**Keywords:** acute myeloid leukaemia (AML), drug sensitivity, epigenetic mutations, isolated trisomies, transcriptomic profiling

## Abstract

Acute myeloid leukaemia (AML) with isolated trisomies (ITs) represents a distinct cytogenetic subgroup with heterogeneous clinical behaviour and incompletely defined molecular features. To explore its genomic and transcriptomic landscape, we performed next‐generation sequencing (NGS) on 14 AML patients harbouring isolated trisomies of chromosomes 8, 9, 10, 13, 14, 21 and 22. RNA sequencing (RNA‐Seq) was conducted on 15 samples, including 12 with IT and 3 cytogenetically normal AML cases (normal karyotype, NK‐AML) serving as controls. Trisomy 8 was most frequent, followed by chromosomes 13, 14 and 21. Recurrent mutations were identified in epigenetic regulators (*DNMT3A*, *IDH1/2*, *ASXL1*, *TET2*). Transcriptomic profiling stratified cases into IT‐8, IT‐21 and IT‐13+22 subgroups. Gene set enrichment analysis (GSEA) revealed shared downregulation of cell cycle‐related pathways (e.g., G2M checkpoint) and subgroup‐specific patterns: oxidative stress and unfolded protein response in IT‐8; epithelial‐mesenchymal transition and oxidative phosphorylation in IT‐21; inflammatory signalling (IL‐6/JAK/STAT, TNF‐α/NF‐κB) in IT‐13+22. A core set of 60 differentially expressed genes (DEGs) was shared, with nine hub genes related to cell cycle (*MCM4*, *CDC7*, *CDC25A*, *DHFR*), proteostasis (*HSPA5*, *DNAJC3*, *CALR*, *HSP90B1*) and inflammation. Drug sensitivity profiling revealed subgroup‐specific vulnerabilities: IT‐8 to DNA damage checkpoint inhibitors, IT‐21 to PLK/mTOR inhibitors and IT‐13+22 to BRAF/EGFR‐targeted agents. These findings highlight AML‐IT heterogeneity and therapeutic potential.

## 1 Introduction

Isolated trisomy (IT) in acute myeloid leukaemia (AML) refers to the presence of an extra chromosome without other major cytogenetic alterations. This common aberration plays a key role in AML pathogenesis and prognosis [1, 2]. While the mechanisms remain unclear, IT may drive leukemogenesis via gene dosage effects or dysregulation of gene networks affecting pathways such as cell cycle and proliferation [1, 2, 3].

Integrating genetic, cytogenetic and clinical data enables more precise AML classification and risk stratification. Specific molecular alterations can further refine patient subgroups and guide therapy [4]. The prognostic impact of isolated trisomies varies by chromosome. Trisomy 8, observed in 10%–15% of AML cases, can occur as an isolated abnormality or in combination with others. It is characterised by marked clinical heterogeneity and is associated with a broad mutational spectrum, including genes involved in DNA methylation (*DNMT3A*), spliceosome regulation (*SRSF2*), signalling pathways (*KRAS, IDH1/2, FLT3, JAK2, KIT*) and transcriptional control (*RUNX1*, *GATA1*, *TET2*), with *ASXL1* mutations carrying strong prognostic implications [5, 6, 7].

Trisomy 9 is rare, with uncertain prognostic value [8]. Trisomy 13 is associated with poor outcomes, low remission rates and mutations in *SRSF2* and *RUNX1* [9]. Trisomy 14, also rare, may represent an early leukemogenic event and is linked to age and mutations in *FLT3*, *KIT*, *JAK2*, *KRAS* and *NPM1* [10]. Trisomy 21, although infrequent in AML, has been reported as a somatic abnormality and is linked to altered haematopoiesis and poor prognosis in some cases [11].

Genomic and transcriptomic profiling via Next‐Generation Sequencing (NGS) and RNA‐Seq are essential for AML risk assessment and therapeutic guidance. Current recommendations support mutation and structural variant detection at diagnosis to inform clinical decisions [12].

Given the limited molecular characterisation of IT‐AML, we investigated its genomic and transcriptomic landscape in a Brazilian cohort with isolated trisomies of chromosomes 8, 9, 10, 13, 14, 21 and 22. NGS was performed on 14 intensively treated patients and RNA‐Seq on 16 samples (13 IT and 3 cytogenetically normal controls). We identified recurrent mutations, transcriptional changes and subgroup‐specific vulnerabilities. Our findings reveal convergent downregulation of cell cycle pathways and distinct transcriptional programs, suggesting shared regulatory mechanisms and potential targets for precision therapy.

## 2 Materials and Methods

### 2.1 Study Design and Patient Eligibility

This study was conducted at the Haematology and Transfusion Medicine Center, University of Campinas and included adult patients (≥ 18 years) newly diagnosed with de novo Acute Myeloid Leukaemia (AML) between 2011 and 2022. Among the 213 diagnosed cases, 89 had a normal karyotype (NK) and 19 had isolated trisomies (IT), i.e., single trisomies—totaling 108 patients included in this study. Clinical and laboratory data were retrospectively collected. At diagnosis, all patients underwent bone marrow immunophenotyping, conventional cytogenetics and molecular testing for *FLT3* (*ITD*/*TKD*) and *NPM1* (types A, B and D) mutations. The study was approved by the local Ethics Committee and informed consent was obtained from all patients or their families. All procedures were conducted in accordance with the Declaration of Helsinki.

### 2.2 Treatment Protocols, Response Criteria and Follow Up

Patients received standard intensive induction chemotherapy with the ‘7+3’ regimen (daunorubicin and cytarabine), followed by high‐dose cytarabine consolidation. Eligibility was based on age, performance status and comorbidities. Transplantation was indicated for intermediate or adverse‐risk patients with a compatible donor, while others received additional consolidation cycles. From 2017, treatment followed the ICAML2015 protocol, including a second induction cycle and risk‐adapted post‐remission strategies. Patients ineligible for intensive therapy were treated with low‐dose cytarabine [13]. Clinical responses were evaluated per standard criteria [14]. Complete remission (CR) required < 5% blasts with hematologic recovery, assessed 28 days post‐treatment. Relapse was defined as ≥ 5% blasts or extramedullary disease. Overall survival (OS) was calculated from induction start to death or last follow‐up. Survival probabilities were estimated up to 5 years, although some patients had longer follow‐up times (exceeding 90 months).

### 2.3 Genomic DNA Extraction, Library Preparation and Sequencing

Genomic DNA was extracted from total bone marrow cells using the DNeasy Blood and Tissue Kit (Qiagen). Leukaemic blasts represented the predominant cell population in this sample as shown in Table S1. DNA quantification was performed using a Qubit Fluorometer. Libraries were prepared with the AmpliSeq Library Plus for Illumina and the AmpliSeq Myeloid Panel (Illumina) (Table S2). Library quality was assessed using TapeStation Analysis Software V3.2. Sequencing was carried out on an Illumina MiSeq platform with v3 reagents (2 × 150 bp) to a minimum depth of 500 ×.

### 2.4 Genomic Analysis

Raw sequencing files were converted to FASTQ format with QC metrics generated throughout the pipeline. QC reports included alignment to the GRCh37/hg19 reference genome and duplicate removal (Q30 > 90%). Variant analysis was performed using the Franklin platform (https://franklin.genoox.com), with data manipulation via R packages dplyr and tidyr. Functional annotations from InterVar and CancerVar assessed clinical relevance [15, 16]. Filtering targeted non‐synonymous variants in coding regions, exon‐flanking sequences, splice sites and 5′‐UTRs (Untranslated Regions), retaining variants with VAF > 3% and read depth > 50. Somatic variants with allele frequency < 0.1% in population databases were considered. Missense variant effects were predicted using SIFT, PolyPhen2, MutationTaster and Mutation Assessor; conservation was assessed via PhastCons and PhyloP. Allele frequencies were obtained from gnomAD and ExAC and variants with CADD > 15 were prioritised. COSMIC (https://cancer.sanger.ac.uk/cosmic) identified recurrent AML mutations and IGV aided in visualisation and artefact filtering. Pathogenicity was evaluated per ACMG guidelines [17], with variants of uncertain significance reviewed manually. A summary of the workflow is shown in (Figure S1).

### 2.5 Exploration IT Mutation Data Using Maftools

Genomic data from 14 IT patients was converted to a MAF file using the ‘annovarToMaf’ function in R and analysed with Maftools (R version 4.5.0) [18], generating visual and statistical results for mutations. Following genomic analysis, transcriptomic analysis was conducted to investigate gene expression and molecular pathway changes associated with IT, aiming to understand its impact on AML biology.

### 2.6 RNA Extraction, Library Preparation and Sequencing (RNA‐Seq)

RNA was extracted from total bone marrow using the RNeasy Mini Kit (Qiagen). Quantification was performed with the NanoDrop‐1000 (Thermo Fisher) and quality assessed via chip‐based electrophoresis on the Bioanalyzer 2100 (Agilent). Libraries were prepared using the Illumina Stranded mRNA Prep Kit, following the manufacturer's protocol, then pooled and sequenced on the NovaSeq S1 platform (200 cycles), using paired‐end 2 × 100 bp reads, with an average depth of 50 million read pairs per library.

### 2.7 RNA‐Seq Data Processing and Analysis

Prior to alignment, abundant sequences, including ribosomal RNA (rRNA), were removed to reduce background and improve quantification accuracy. Reads were then aligned to the reference genome ( *Homo sapiens* , hg19) using STAR (Version 2.6.1a) [19], allowing for the detection of splice junctions and gene fusions. Manta is then employed to identify potential gene fusions by analysing discordant read pairs and split reads. Finally, transcript expression is quantified with Salmon (Version 0.11.2) [20], providing highly accurate estimates of gene and transcript abundance.

### 2.8 Differentially Expressed Genes Analysis Between IT and NK‐AML

Differential expression analysis was conducted using edgeR, DESeq2, and Limma in R, selected for their complementary sensitivities to data distribution, enhancing result robustness [21]. Raw counts were normalised and low‐expression genes were filtered. Differentially expressed genes (DEGs) between trisomy groups (IT‐8, IT‐21, and IT‐13+22) and NK were identified using |Log2FC| > 1 and *p* < 0.05. Three NK‐AML cases were selected as internal reference controls for this analysis based on their genomic characterisation (summarised in Table S3). PCA (Principal Component Analysis) performed on log2‐transformed, normalised RNA‐seq expression values indicated similar profiles for IT‐13 and IT‐22, which were therefore analysed together.

Raw counts were normalised and low‐expression genes were filtered. For visualisation purposes (heatmaps and expression plots), transcripts per million (TPM) values were also calculated. A final DEG list was compiled by integrating genes commonly identified or meeting *p* < 0.05 or False Discovery Rate (FDR) < 0.05 across methods. Hierarchical clustering (Euclidean distance) and heatmaps (ComplexHeatmap—Release 2.24.1, InteractiveComplexHeatmap—Release 1.16.0, and pheatmap) were used to assess expression patterns and subgroup profiles [22, 23].

### 2.9 Functional and Pathway Enrichment Analysis

Functional enrichment analysis was first performed using the GSEA software (Version 4.3.3), and results were independently confirmed with the FGSEA (Version 1.34.2) and clusterProfiler (Version 4.16.0) packages in R [24, 25, 26]. Hallmark gene sets and KEGG pathways were evaluated to ensure robustness across methods. Significance was determined via 1000 permutations, weighted statistics and Signal2Noise ranking. Gene sets with 10–500 genes, FDR < 25% and *p* < 0.05 were considered enriched. Normalised Enrichment Scores (NES) values categorised enrichment as positive or negative. DEG filtering used R tools (dplyr, plyr, readr, openxlsx) by cross‐referencing with MSigDB‐enriched sets. Gene Ontology (GO) analysis via DAVID (Version v2024q4, https://david.ncifcrf.gov) evaluated biological process (BP), cellular component (CC) and molecular function (MF), with Benjamini‐Hochberg‐adjusted *p* < 0.05.

### 2.10 Drug Sensitivity Analysis

Drug sensitivity was predicted using the OncoPredict R package (https://www.r‐project.org/; [27]), which analyzes gene expression profiles from the IT and NK groups. The **GDSC2 dataset** was used as the reference for both drug response and gene expression. Testing gene expression data were log2‐transformed prior to analysis and batch effects were corrected using empirical Bayes methods. Low‐variance genes were filtered according to the OncoPredict recommendations. Predictions were generated using the built‐in calcPhenotype() function, and no internal training of the model was performed. Predicted IC50 values for each drug were then statistically compared between groups using the Wilcoxon rank‐sum test and significant drugs were visualised with boxplots.

### 2.11 Statistical Analysis

Clinical data for NK and IT patients were summarised as frequencies and medians. Group comparisons used Fisher's exact test for categorical variables and the Kruskal–Wallis test for comparisons involving more than two groups. A significance level of *p* < 0.05 was considered, and P‐values were adjusted for multiple testing using the Benjamini–Hochberg (BH) method where applicable. Overall survival (OS) was estimated using Kaplan–Meier curves and the log‐rank test, including only patients treated with induction intensive chemotherapy. Survival analyses and plots were generated using the *survminer* and *survival* packages in R (version 4.3.3, 2024‐02‐29; https://www.r‐project.org/).

## 3 Results

### 3.1 Patient Characteristics

Clinical data were analysed from 108 AML patients, with a mean age of 61 years (range: 19–93). Clinical characteristics, ELN 2017 risk stratification and outcomes are summarised in Table 1. No significant differences were found between the NK and IT groups regarding concordance. A total of 71 patients (65.7%) received induction therapy, corresponding to 57 (64.0%) in the NK group and 14 (73.7%) in the IT group. Only 20 patients (18.5%) underwent allogeneic stem cell transplantation (Allo‐SCT), with a higher proportion in the NK group. Complete remission was achieved in 41 patients (57.7%). The median overall survival (OS) was 4.3 months for the NK group and 5.5 months for the IT group, though these differences were not statistically significant (Figure S2).

**TABLE 1:** Clinical features of NK‐AML and IT‐AML patients.

Patient characteristics	Patients, no. (%)	NK‐AML *n* = 89	IT‐AML *n* = 19	*p*
Gender				
Male	59 (54.6)	45 (50.6)	14 (37.7)	0.790^a^
Female	49 (45.4)	44 (49.4)	5.0 (26.3)	
Age (y)				
Median (range)	61 (19–93)	61 (21–83)	54 (19–93)	0.351^**b**^
10–40	15 (13.9)	10 (11.2)	5.0 (26.3)	
41–60	38 (35.2)	33 (37.1)	5.0 (26.3)	
> 60	55 (50.9)	46 (51.7)	9.0 (47.4)	
Haemoglobin, (g/dL)				
Median (range)	8.5 (4.6–14.9)	8.5 (4.6–11.5)	8.9 (5.3–14.9)	0.347^**b**^
0–7	20 (18.5)	18 (20.2)	2.0 (10.5)	
7–10	71 (65.7)	59 (66.3)	12 (63.2)	
10–12	17 (15.7)	12 (13.5)	5.0 (26.3)	
Na	—	—	—	
WBC count, (×10^9^/L)				
Median (range)	18.4 (1–371)	18.2 (1–371)	19 (1–206)	0.672^**b**^
0–4	28 (25.9)	22 (24.7)	6.0 (31.6)	
4–10	15 (13.9)	13 (14.6)	2.0 (10.5)	
> 10	65 (60.2)	54 (60.7)	11 (57.9)	
Na	—	—	—	
Percentage of BM blasts				
Median (range)	66.7 (2.5–98.5)	68.5 (20–98.5)	50 (2.5–94)	0.140^**b**^
< 40	28 (25.9)	20 (22.5)	8.0 (42.1)	
40–70	29 (26.9)	25 (28.1)	4.0 (21.1)	
> 70	51 (47.2)	44 (49.4)	7.0 (36.8)	
Na	—	—	—	
Platelet count, (×10^9^/L)				
Median (range)	52 (6.0–537)	53 (6.0–537)	40 (11–497)	0.417^**b**^
100–150	26 (24.1)	22 (24.7)	4.0 (21.1)	
20–100	70 (64.8)	59 (66.3)	11 (57.9)	
0–20	12 (11.1)	8.0 (9.0)	4.0 (21.1)	
Na	—	—	—	
NPM1, *n* (%)				
Mutated	31 (28.7)	29 (32.6)	7.0 (36.8)	0.292^a^
Wild‐type	47 (43.5)	36 (40.4)	8.0 (42.1)	
Na	30 (27.8)	24 (27.0)	4.0 (21.1)	
FLT3‐ITD, *n* (%)				
Mutated	20 (18.5)	17 (19.1)	3.0 (15.8)	0.517^**a**^
Wild‐type	56 (51.9)	48 (53.9)	8.0 (42.1)	
Na	32 (29.6)	24 (27.0)	8.0 (42.1)	
NPM1‐FLT3, *n* (%)				
NPM1+/FLT3+ and NPM1−/FLT3−	59 (75.6)	49 (75.4)	10 (76.9)	0.029^**a**^
NPM1+/FLT3−	14 (17.9)	14 (21.5)	0.0 (0.0)	
NPM1−/FLT3+	5.0 (6.4)	2.0 (3.1)	3.0 (23.1)	
ELN risk				
Favourable	20 (18.5)	20 (22.5)	0.0 (0.0)	0.029^**a**^
Intermediate	83 (76.9)	66 (74.2)	17 (89.5)	
Adverse	2.0 (1.9)	1.0 (1.1)	1.0 (5.3)	
Na	3.0 (2.8)	2.0 (2.2)	1.0 (5.3)	
Intensive chemotherapy				
Yes	**71 (65.7)**	57 (64.0)	14 (73.7)	0.595^**a**^
No	37 (34.3)	32 (36.0)	5.0 (26.3)	
Allo SCT				
Yes	20 (18.5)	14 (15.7)	6.0 (31.6)	0.116^**a**^
No	88 (81.5)	75 (84.3)	13 (68.4)	
CR, rate (%)				
Yes	41 (57.7)	31 (54.4)	10 (71.4)	0.251^**a**^
No	30 (42.3)	26 (45.6)	4.0 (28.6)	
Na	—	—	—	
Overall survival				
Median (months) 95% CI	4.59 (0.06–92.8)	4.3 (0.06–92.8)	5.51 (0.46–64.6)	0.320^**c**^

Abbreviations: Allo‐SCT, allogenic stem cell transplant; BM, bone marrow; CI, confidence interval; CR, complete remission; ELN^2017^, European Leukaemia Net; Na, not applicable; WBC, white blood cell count.

^**a**^ Fisher exact probability test.

^**b**^ Kruskal–Wallis one‐way test.

^**c**^ Log‐rank test.

### 3.2 Genomic Characterisation of Isolated Trisomies in AML

**FIGURE 1:**
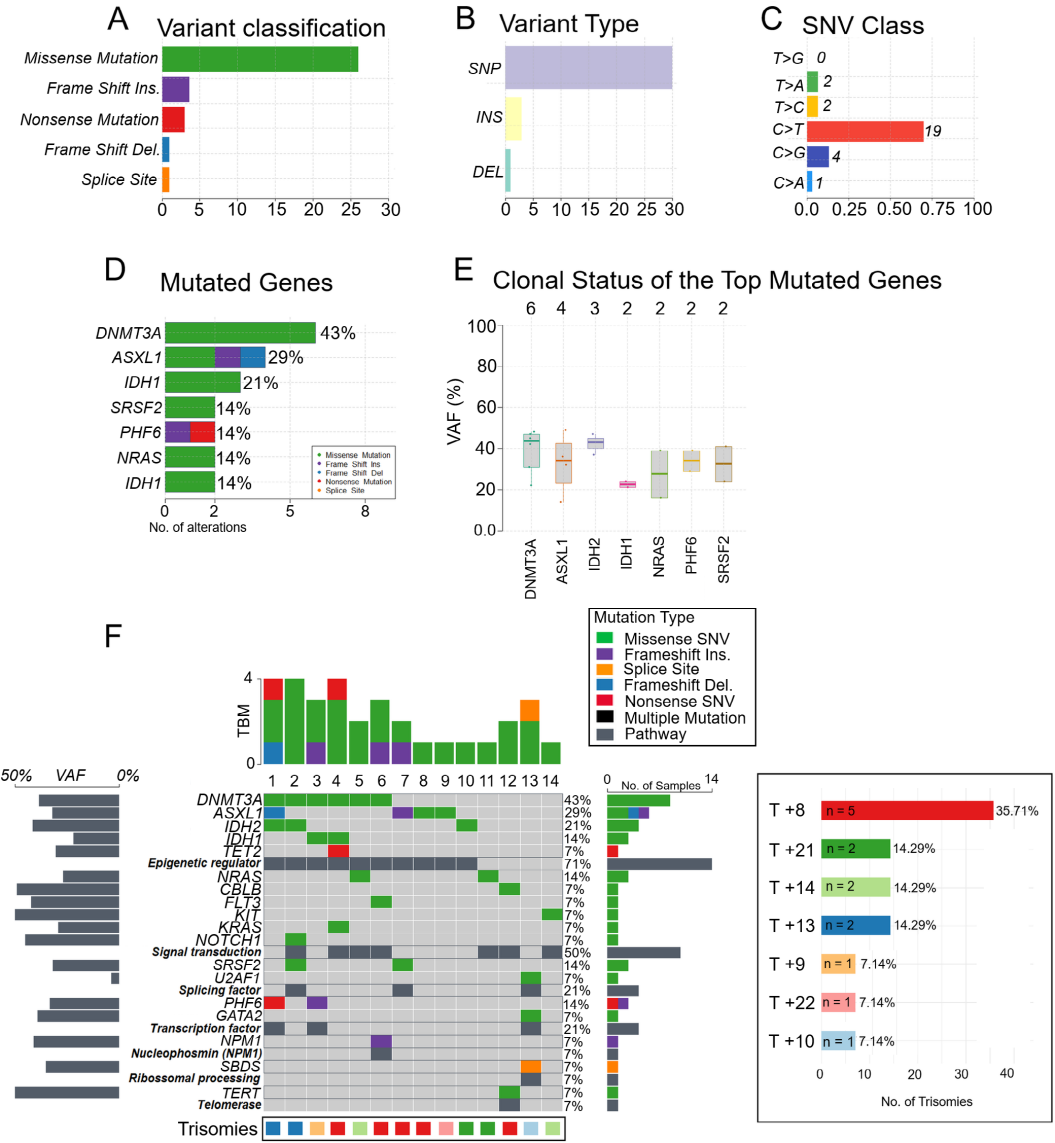
Summary of mutations found in 14 individuals with IT. (A) Distribution of variant classifications, highlighting missense mutations as the most common type. (B) Variant types were identified across the cohort, categorised as SNPs, insertions (INS) and deletions (DEL). (C) Single nucleotide variant (SNV) substitution classes, highlighting the most common transition (C>T). (D) Frequency and type of mutations in the top seven most frequently mutated genes, with *DNMT3A* and *ASXL1* being the most altered. (E) Clonal status of recurrently mutated genes based on variant allele frequency (VAF), suggesting that mutations in *DNMT3A* and *ASXL1* are likely clonal, while others like *IDH1* and *SRSF2* may be subclonal in some cases. (F) An oncoplot showing the heat map of variant classification for the top 18 genes across 14 trisomies. The X‐axis shows different samples and the Y‐axis shows the top 18 genes. Genes are grouped by function in the pathway in which they act. The most relevant functions were epigenetic regulators, 71% (*DNMT3A*, *IDH2‐1*, *ASXL1*, *TET2*), signal transduction, 50% (*NRAS*, *CBLB*, *FLT3*, *KIT*, *KRAS*, *NORCH1*), splicing factor and transcription factor, 21%. Allele frequencies for each gene are displayed on the left, which estimates the clonal status of the top mutated genes. Variants noted as multi‐hit are identified in genes mutated more than once in the same patient sample. The top bar graph indicates tumor mutation burden (TMB) per sample, while the bar plot on the left shows the average VAF for each gene, helping estimate clonal status. The bar chart on the right displays the frequency of each isolated trisomy among the cohort, with trisomy 8 being the most prevalent (35.7%), followed by trisomies 13, 14 and 21 (each 14.2%).

In Figure 1A–E, we present a summary of the somatic IT mutations identified in the 14 patients in our cohort. The variant classification reveals that the most abundant mutations are missense mutations. Single nucleotide variants (SNVs) are the most prevalent type, with transitions being more common than transversions. The graphical representation in Figure 1F, known as an oncoplot, highlights the 18 most frequently mutated genes in the samples analysed. In 10 patients (71%), molecular changes were identified in genes related to the regulation of the epigenetic pathway, including *DNMT3A* (43%), *ASXL1* (29%), *IDH2* (21%), *IDH1* (14%) and *TET2* (7%).

Our cohort identified trisomy 8 as the most common, observed in 5 cases (35.7%). Additionally, trisomies of chromosomes 21, 14 and 13 were found in 14.3% of cases, with two patients presenting each. Trisomies of chromosomes 9, 22 and 10 were observed in 7.14% of cases, with one patient presenting each (Figure 1F). Detailed information on the mutations in each patient can be found in Table 2.

**TABLE 2:** Genetic variants identified in patients with isolated trisomy.

ID	Gender/Age	Trisomy	Mutated gene	Location	HGVSc	HGVSp	Type	VAF	Depth	dbSNP	COSMIC ID	ACMG classifier
1	F‐70	Trisomy +13	*IDH2* (NM_002168.4)	Chr:15; Exon‐4	c.419G>A	p.Arg140Gln	Missense	0.47	2078	rs121913502	COSM41590*	Pathogenic
			*ASXL1* (NM_015338.6)	Chr:20; Exon‐12	c.1900_1922del	p.E630Rfs*15	Indel	0.32	1618	rs766433101	COSM3720455*	Likely pathogenic
			*PHF6* (NM_001015877)	Chr:X; Exon‐10	c.975C>G	p.Tyr325Ter	Nonsense	0.39	327	—	—	Uncertain significance
			*DNMT3A* (NM_175629)	Chr:2; Exon‐23	c.2645G>A	p.Arg882His	Missense	0.45	1526	rs147001633	COSM442676*	Pathogenic
2	M‐61	Trisomy +13	*IDH2* (NM_002168.4)	Chr:15; Exon‐4	c.419G>A	p.Arg140Gln	Missense	0.43	2349	rs147001633	COSM41590*	Pathogenic
			*DNMT3A* (NM_022552.5)	Chr:2; Exon‐23	c.2645G>A	p.Arg882His	Missense	0.45	1466	rs147001633	COSM442676*	Pathogenic
			*SRSF2* (NM_003016.4)	Chr:17; Exon‐1	c.284C>G	p.Pro95Arg	Missense	0.24	126	rs751713049	COSM211661*	Pathogenic
			*NOTCH* (NM_017617)	Chr:9; Exon‐16	c.2495C>T	p.Pro832Leu	Missense	0.46	2416	rs559917218	—	Uncertain significance
3	M‐65	Trisomy +9	*DNMT3A* (NM_175629)	Chr:2; Exon‐2	c.2644C>T	p.Arg882Cys	Missense	0.22	953	rs377577594	COSM53042*	Likely pathogenic/Pathogenic
			*IDH1* (NM_005896)	Chr:2; Exon‐4	c.394C>T	p.Arg132Cys	Missense	0.24	308	rs121913499	COSM28747*	Likely pathogenic/Pathogenic
			*PHF6* (NM_001015877)	Chr:X; Exon‐9	c.70dupA	p.Asn23fs	Indel	0.29	500	—	COSM4385769*	Likely pathogenic
4	M‐54	Trisomy +8	*KRAS* (NM_033360.4)	Chr:12; Exon‐3	c.179G>A	p.Gly60Asp	Missense	0.30	1908	—	COSV55548621*	Pathogenic
			*IDH1* (NM_005896.4)	Chr:2; Exon‐4	c.394C>T	p.Arg132Cys	Missense	0.21	1872	rs121913499	COSV61615256*	Likely pathogenic/Pathogenic
			*TET2* (NM_001127208.3)	Chr:4; Exon‐11	c.5479A>T	p.Lys1827Ter	Nonsense	0.31	3170	—	COSV54404533*	Uncertain significance
			*DNMT3A* (NM_022552.5)	Chr:2; Exon‐8	c.875 T>C	p.Ile292Thr	Missense	0.46	1168	rs777306476	COSV53056599*	Pathogenic
5	M‐67	Trisomy +14	*NRAS* (NM_002524.5)	Chr:1; Exon‐2	c.34G>A	p.Gly12Ser	Missense	0.16	1612	rs121913250	COSM53042*	Pathogenic
			*DNMT3A* (NM_022552.5)	Chr:2; Exon‐23	c.2644C>T	p.Arg882Cys	Missense	0.31	1542	rs377577594	COSM53042*	Pathogenic
6	F‐38	Trisomy +8	*DNMT3A* (NM_175629)	Chr:2; Exon‐23	c.2645G>A	p.Arg882His	Missense	0.42	1327	rs147001633	COSV53036153*	Pathogenic
			*NPM1* (NM_001355010)	Chr:5; Exon‐7	c.478_479insTCTG	p.Leu160fs	Indel	0.42	192	rs587776806	—	Likely pathogenic
			*FLT3* (NM_004119)	Chr:13; Exon‐20	c.2503G>T	p.Asp835Tyr	Missense	0.43	897	—	COSV54042116*	Pathogenic
7	M‐64	Trisomy +8	*SRSF2* (NM_003016)	Chr:17; Exon‐1	c.284C>G	p.Pro95Arg	Missense	0.41	208	rs751713049	COSV57969809*	Likely pathogenic/Pathogenic
			*ASXL1* (NM_001363734)	Chr:20; Exon‐11	c.1744dupG	p.Gly581fs	Indel	0.36	565	rs1085307856	—	Likely pathogenic/Pathogenic
8	F‐29	Trisomy +8	*ASXL1* (NM_001363734)	Chr:20; Exon‐7	c.683C>T	p.Pro228Leu	Missense	0.14	68	—	—	Pathogenic
9	M‐46	Trisomy +22	*ASXL1* (NM_001363734)	Chr:20; Exon‐12	c.3727C>G	p.Leu1243Val	Missense	0.49	767	rs747267907	—	Pathogenic
10	F‐49	Trisomy +21	*IDH2* (NM_001290114)	Chr:15; Exon‐2	c.419G>A	p.Arg140Gln	Missense	0.37	640	rs121913502	COSM41590*	Pathogenic
11	F‐31	Trisomy +21	*NRAS* (NM_002524)	Chr:1; Exon‐2	c.35G>A	p.Gly12Asp	Missense	0.39	748	rs121913237	COSM564*	Pathogenic
12	M‐47	Trisomy +8	*TERT* (NM_198253.3)	Chr:5; Exon‐3	c.1594G>A	p.Ala532Thr	Missense	0.50	639	rs776459827	—	Uncertain significance
			*CBLB* (NM_001321796)	Chr:3; Exon‐11	c.1771C>T	p.Arg591Trp	Missense	0.50	2264	rs372086643	COSM5989837*	Uncertain significance
13	M‐29	Trisomy +10	*GATA2* (NM_032638.5)	Chr:3; Exon‐5	c.1085G>A	p.Arg362Gln	Missense	0.40	2729	rs867160952	COSM87004*	Pathogenic
			*SBDS* (NM_016038.4)	Chr:7; Exon‐2	c.258+2 T>C	—	Splicing	0.36	2120	rs113993993	COSM5020081	Pathogenic
14	M‐28	Trisomy +14	*KIT* (NM_000222)	Chr:4; Exon‐17	c.2447A>T	p.Asp816Val	Missense	0.50	1081	rs121913507	COSM1314*	Pathogenic

*Note:* The accession number (NM_) is the National Center for Biotechnology Information database, Reference Sequence (https://www.ncbi.nlm.nih.gov/RefSeq); HGVSc, Human Genome Variation Society Transcript Nomenclature (for coding region); HGVSp, Human Genome Variation Society protein nomenclature; VAF, variant allele frequency is the fraction of the variant allele, that is, the proportion of the mutated allele charge about the number of normal cells; Depth of coverage is the average number of mapped reads at a given locus. ACMG classifier, The American College of Medical Genetics and Genomics: Pathogenic or Likely Pathogenic, VUS: variant of uncertain significance. (*) COSM ID. representative for mutation in Haematopoietic tissue.

### 3.3 Overall Mutations Description

Co‐occurring mutations were observed in patients with trisomies: 8 (patients 4, 6, 7); 9 (patient 3); 10 (patient 13); 13 (patients 1, 2); and 14 (patient 5) (Figure 1F and Table 2). These mutations often co‐occur and may be functionally related, potentially cooperating to drive pathological processes and predict worse prognosis [28, 29]. Similar patterns of co‐occurring mutations in myeloid diseases have been reported, including *DNMT3A*, *TET2* and *SF3B1* in MDS and VEXAS syndrome [30]. We analysed the VAFs of co‐occurring mutations in patients harbouring two or more mutations to assess their clonal representation. Mutations with VAFs greater than 0.3 were considered to reside in dominant clones, as their allele frequencies were higher than those of the other mutations identified in the same patient. This approach allowed us to highlight mutations most likely contributing to leukemogenesis within major clonal populations (Table 1).

In this study, molecular heterogeneity is defined as the presence of distinct driver mutations within isolated trisomies, affecting genes involved in epigenetic regulation, RNA splicing, signalling pathways, and transcriptional programs. This highlights the coexistence of multiple genetically and functionally diverse cell populations that may contribute to disease progression [31, 32]. In trisomy 8, co‐occurring pathogenic mutations were found in genes such as *DNMT3A*, *KRAS*, *FLT3*, *NPM1*, *SRSF2* and *ASXL1*, suggesting molecular heterogeneity with multiple genetic pathways affected [6].

In the single trisomy 9 case, mutations in *DNMT3A*, *IDH1* and *PHF6* may play a crucial role in disease pathogenesis. Similarly, in trisomy 10, mutations appeared in *GATA2* and *SBDS*. Co‐occurring mutations in *IDH2* and *DNMT3A* were found in two trisomy 13 cases. For trisomy 14, high‐risk mutations were seen in *NRAS*, *DNMT3A* and *KIT* [10]. Unique mutations were identified in trisomy 21 (*IDH2*, *NRAS*) and trisomy 22 (*ASXL1*), all linked to pathogenic effects and unfavourable outcomes [11, 33].

### 3.4 Transcriptome Sequencing Quality and Mapping

Reads were aligned to the human reference genome (GRCh19), with an average mapping rate of 94.32% across samples (range: 89.65%–96.45%). Multi‐mapped reads varied between 3.24% and 13.74%, while duplication levels were consistently low (0.12%–4.41%), indicating good alignment specificity and library complexity. Mean fragment lengths ranged from 166.98 bp to 187.47 bp, consistent with expected RNA‐seq profiles. Mitochondrial reads accounted for 8.49%–19.64% of total reads, while ribosomal content remained low (1.11%–3.59%). Gene detection at a depth of ≥ 10 reads (Genes 10X) ranged from 10,685 to 12,803 across samples, reflecting robust transcriptome coverage (Table S4).

### 3.5 Analysis of DEGs and Signalling Pathway Enrichment in Trisomy

**FIGURE 2:**
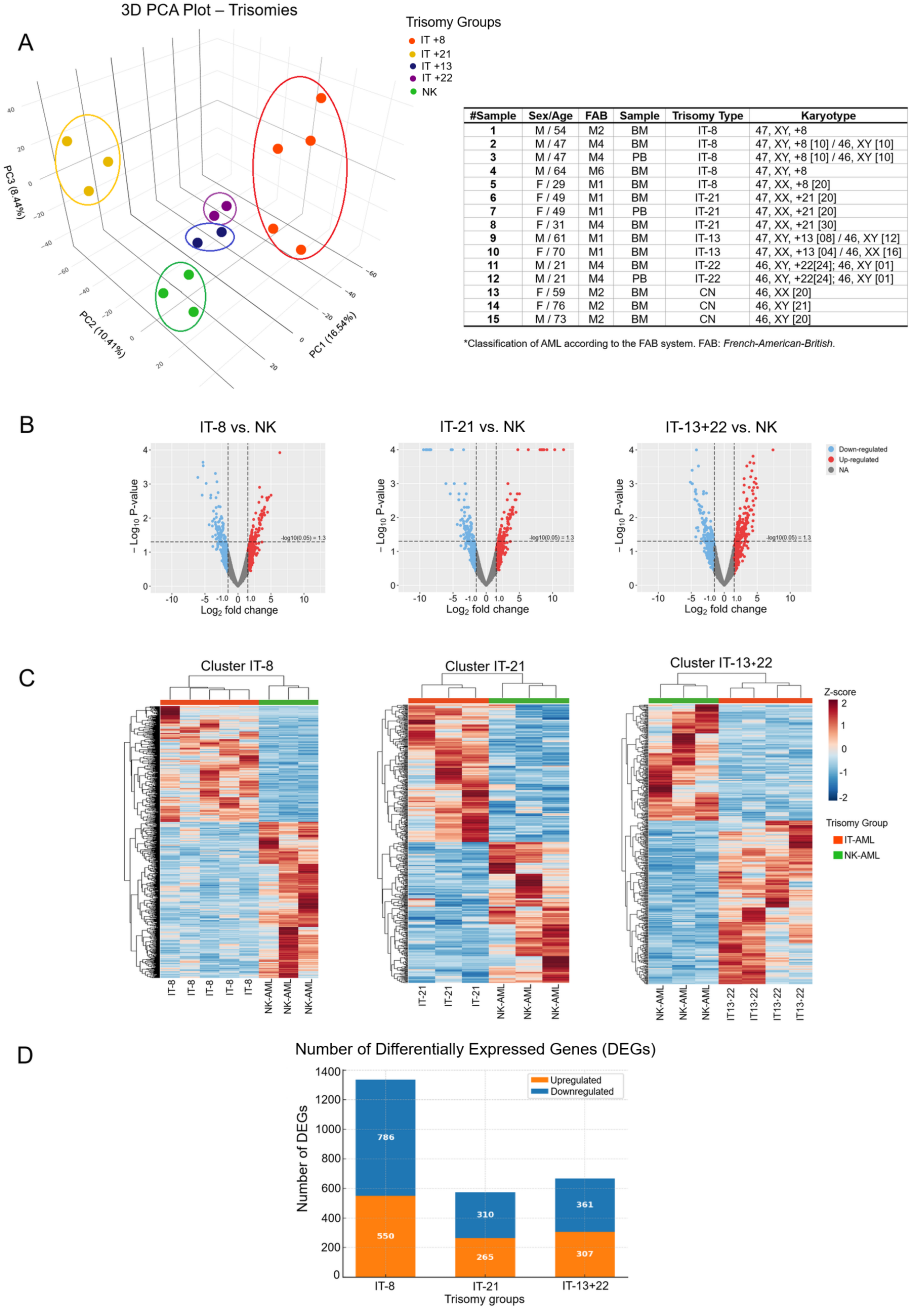
Differential Gene Expression Analysis Between Trisomies and Control Group. (A) Three‐dimensional Principal Component Analysis (PCA) illustrating the separation between IT‐8, IT‐21, IT‐13+22 and the control group NK. Each dot represents a sample, coloured according to its genetic condition, with ellipses indicating sample clustering based on principal variance. (B) Volcano plots displaying differentially expressed genes in each comparison between trisomies and the control group NK. Upregulated genes are shown in red, while downregulated genes appear in blue. The significance threshold was set at log2 fold change ≥ |1.0| and *p*‐value < 0.05. (C) Heatmaps representing differential gene expression in specific clusters for each trisomy (IT‐8, IT‐21 and IT‐13+22) compared to the NK group. The colour scale ranges from blue (lower expression) to red (higher expression) and hierarchical clustering analysis using Euclidean distance and *Z*‐score normalisation highlights distinct gene regulation patterns among the groups. To minimise the influence of sample‐specific variability, all DEG analyses incorporated variance shrinkage, normalisation, and cross‐method concordance across *DESeq2*, *edgeR*, and *limma‐voom*. Therefore, the expression differences observed in the heatmaps represent consistent group‐level transcriptional trends rather than individual‐driven effects. (D) Number of DEGs in samples from patients with IT. The stacked bar chart shows the number of upregulated (orange) and downregulated (blue) genes in the IT‐8, IT‐21 and IT‐13+22 groups. The IT‐8 exhibited the highest number of DEGs, totaling 1336 genes, followed by the IT‐13+22 (668 genes) and the IT‐21 group (575 genes).

Principal component analysis (PCA) was applied to visualise the global transcriptomic variation between IT and normal karyotype (NK) samples. The 3D PCA plot revealed distinct spatial segregation of samples according to trisomy type, indicating specific gene expression signatures for each group (Figure 2A). The first three principal components explained 16.54%, 10.41%, and 8.44% of the total variance, respectively. IT‐8, IT‐21, and the cluster formed by IT‐13 and IT‐22 occupied separate regions in the 3D space, supporting the presence of trisomy‐specific transcriptional profiles. The proximity between IT‐13 and IT‐22 samples along the main components justified their joint evaluation, IT‐13+22 in subsequent differential expression analyses.

Volcano plots and heatmaps (Figure 2B,C) highlighted distinct transcriptional signatures for each trisomy group. DEGs were visualised using a red‐to‐blue colour gradient, where red indicates upregulated genes and blue indicates downregulated genes. IT‐8 displayed the broadest DEG distribution, suggesting more extensive transcriptional deregulation. IT‐21 exhibited a more balanced expression shift, while IT‐13+22 showed an intermediate pattern with defined up‐ and downregulated gene sets. In total, 2579 DEGs were identified. IT‐8 presented 1336 DEGs (550 upregulated, 786 downregulated); IT‐21 had 575 DEGs (265 up, 310 down) and IT‐13+22 showed 668 DEGs (307 up, 361 down) (Figure 2D).

### 3.6 Distinct Transcriptional Programs and Pathway Dysregulation Across Trisomy Subtypes

**FIGURE 3:**
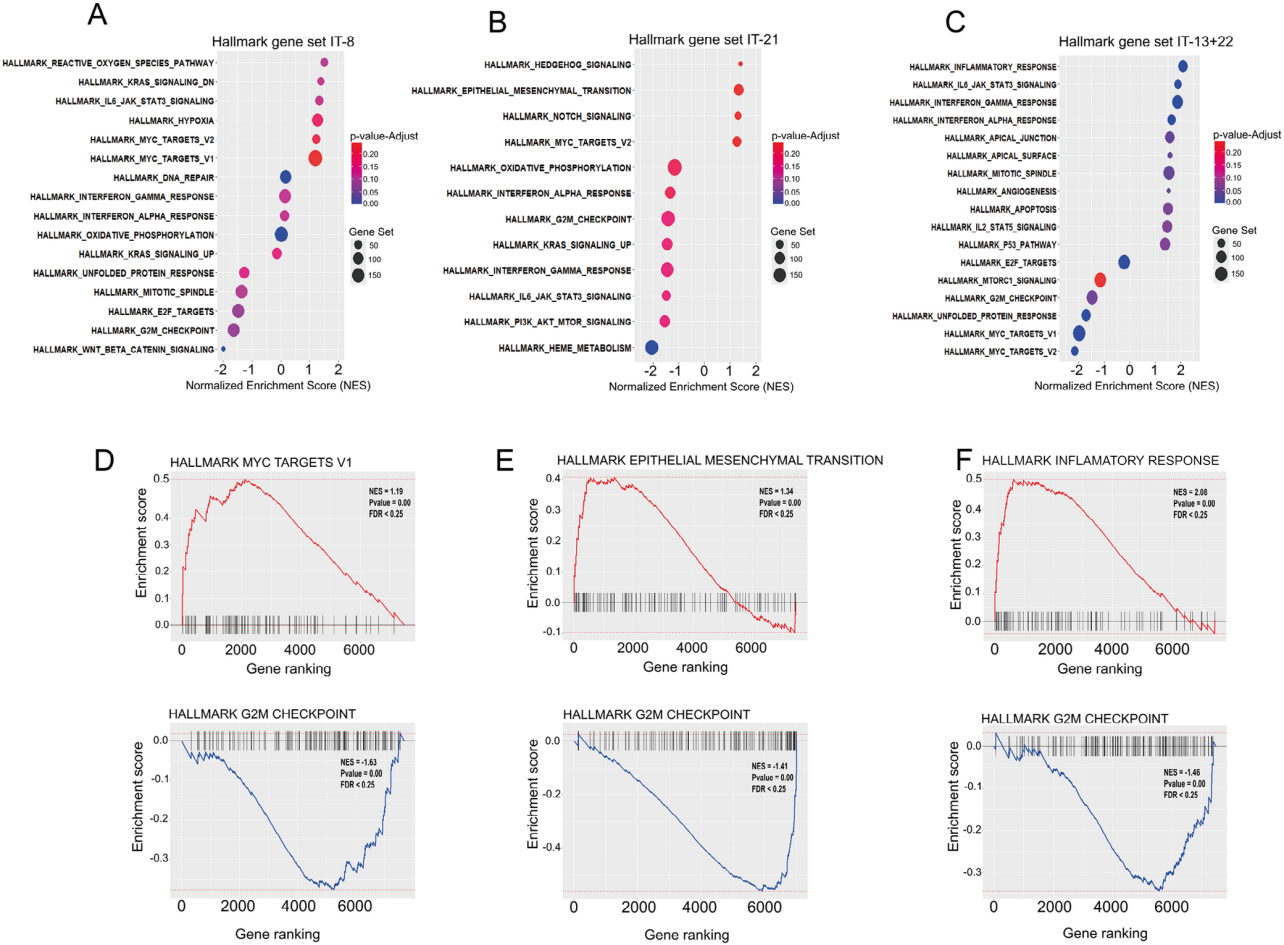
Gene Set Enrichment Analysis (GSEA) of Hallmark pathways in different trisomy groups. (A–C) Dot plots showing significantly enriched Hallmark gene sets (adjusted *p*‐value < 0.25) based on differentially expressed genes in patients with isolated trisomy 8 (A), trisomy 21 (B) and combined trisomies 13 and 22 (C). The *x*‐axis represents the Normalised Enrichment Score (NES); the colour gradient corresponds to the adjusted *p*‐value and dot size reflects the number of genes within each pathway. (D–F) Representative enrichment plots of selected Hallmark gene sets with both positive and negative NES values for each trisomy group. (D) In the IT‐8 group, *MYC_TARGETS_V1* and *G2M_CHECKPOINT* are shown. (E) In the IT‐21 group, *EPITHELIAL_MESENCHYMAL_TRANSITION* and *G2M_CHECKPOINT* are highlighted. (F) For the IT‐13+22 group, enrichment plots illustrate *INFLAMMATORY_RESPONSE* and *G2M_CHECKPOINT*. NES, nominal *p*‐value and FDR are indicated in each plot. GSEA was performed using the clusterProfiler R package (available at: https://guangchuangyu.github.io/software/clusterProfiler/).

Enrichment analysis using Hallmark gene sets revealed distinct transcriptional programs across the trisomy groups, highlighting key pathways either repressed or activated in each context. The comparative dot plots (Figure 3A–C) illustrate the top enriched pathways in IT‐8, IT‐21 and IT‐13+22 groups, based on NES. Negatively enriched gene sets (negative NES) reflect downregulated biological programs, while positively enriched sets (positive NES) indicate transcriptional activation. The full enrichment results are presented separately in Tables S4A (IT‐8), S4B (IT‐21) and S4C (IT‐13+22).

One of the most significantly affected pathways across all trisomy groups was the G2M Checkpoint, which consistently exhibited negative normalised enrichment scores (NES), indicating marked downregulation (Figure 3A). This trend suggests a disruption in cell cycle progression and mitotic control—a common hallmark of chromosomal instability in leukaemia [34]. Additionally, the *MYC* Targets V1 gene set also showed negative NES in both IT‐8 and IT‐13+22, reinforcing the hypothesis of transcriptional repression of *MYC*‐regulated genes, which are essential for cellular proliferation and metabolic regulation [35].

In contrast, several hallmark pathways showed strong positive enrichment. In IT‐8, there was prominent upregulation of stress‐related and signalling pathways, including Reactive Oxygen Species (ROS) Pathway, *KRAS* Signalling and the Unfolded Protein Response, suggesting increased oxidative and proteotoxic stress, as well as aberrant activation of oncogenic signalling cascades [36, 37, 38]. These alterations may reflect cellular responses to aneuploidy and genomic instability, which are not only characteristic of trisomy 8, but also recognised as key hallmarks of cancer.

The IT‐21 group exhibited a distinct enrichment profile (Figure 3B). Notably, the Epithelial‐Mesenchymal Transition (EMT) pathway was among the top positively enriched gene sets, with a high NES, suggesting increased plasticity and potential acquisition of stem‐like or migratory features by leukaemic cells. Furthermore, upregulation of Hedgehog Signalling and mTORC1 Signalling in IT‐21 indicates dysregulation of developmental and growth pathways critical for haematopoietic stem cell maintenance and leukemogenesis. The enrichment of Oxidative Phosphorylation in this group highlights alterations in mitochondrial metabolism, potentially reflecting a shift in energy production mechanisms and cellular adaptation to increased metabolic demands.

The IT‐13+22 subgroup also displayed a unique pattern of pathway enrichment (Figure 3C). Most notably, the Inflammatory Response gene set was strongly upregulated, with one of the highest positive NES values among all comparisons, suggesting robust activation of pro‐inflammatory transcriptional programs. This was accompanied by enrichment in IL6–JAK–STAT3 Signalling, TNF‐alpha Signalling via NF‐κB and Interferon Responses (alpha and gamma), collectively indicating a transcriptional landscape dominated by inflammatory signalling.

Additional enrichment in Apoptosis and Angiogenesis pathways suggests that IT‐13+22 may also engage mechanisms related to cell death and vascular remodelling, potentially influencing leukaemic cell survival and interactions with the microenvironment. Notably, mTORC1 Signalling was also activated in this group, reinforcing the theme of deregulated growth and metabolic control. Despite these inflammatory and proliferative profiles, the negative enrichment of G2M Checkpoint and *MYC* Targets V1 suggests paradoxical repression of key cell cycle drivers, possibly reflecting complex regulatory feedback or compensatory mechanisms specific to this trisomic context.

Enrichment plots (Figure 3D–F) illustrate representative examples of both positively and negatively enriched pathways across trisomy groups, highlighting their molecular heterogeneity, here defined as variability in the biological processes affected. While all groups show evidence of cell cycle dysregulation, each displays distinct alterations in signalling, metabolic, and immune‐related pathways, which may underlie their differing clinical behaviours.

### 3.7 Integrated Analysis of Trisomies Reveals a Common Molecular Core and Characteristic Cellular Dysfunctions

**FIGURE 4:**
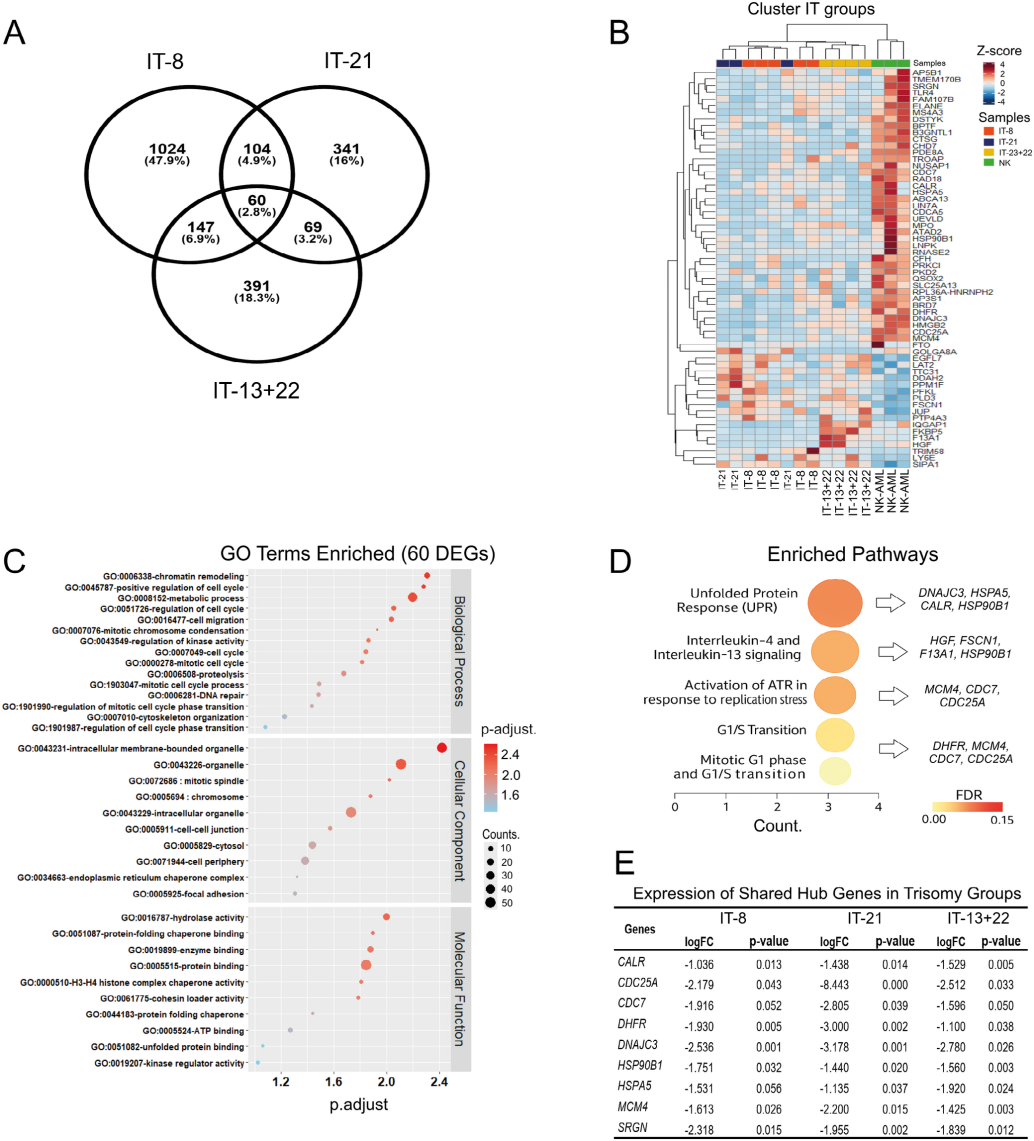
Transcriptomic characterisation of DEGs shared among trisomy groups. (A) Venn diagram showing the overlap of DEGs identified in the IT‐8, IT‐21 and IT‐13+22 groups. A total of 60 genes were commonly identified among the three groups. (B) Heatmap representing the expression levels of the 60 shared genes, with hierarchical clustering of samples based on trisomy type. The colour scale indicates gene expression *Z*‐scores. (C) Gene Ontology (GO) enrichment analysis of the 60 common DEGs, highlighting significantly associated terms in biological processes, cellular components and molecular functions. Dot size represents the number of genes associated with each term and colour indicates the adjusted *p*‐value (*p*.adjust). (D) Enriched biological pathways among the genes shared by the trisomy groups, including unfolded protein response (UPR), interleukin signalling and cell cycle transitions. Circle size reflects the number of genes per pathway and colour indicates FDR. (E) Expression of (9) shared hub genes in trisomy subtypes.

We identified a core set of 60 DEGs shared across the trisomy groups (IT‐8, IT‐21 and IT‐13+22), which exhibited consistent expression patterns distinct from those observed in control samples (Figure 4A,B and Table S5). Moreover, our analyses revealed significant enrichment of key GO terms such as chromatin remodelling (GO:0006333), cell cycle regulation (GO:0001726) and DNA repair (GO:0006941) (Figure 4C and Table S6). These findings suggest that trisomies have a profound impact on core processes involved in genome maintenance.

At the cellular level, we observed striking alterations in essential structural components, including the mitotic spindle (GO:0072685), focal adhesions (GO:0005925) and intracellular organelles (GO:0043229), which may compromise accurate chromosome segregation. Molecular analysis also revealed significant upregulation in protein binding (GO:0005516), ATP interaction (GO:0009824) and molecular chaperone activity (GO:0041815), suggesting compensatory mechanisms aimed at maintaining proteostasis.

Reactome pathway analysis revealed two major axes of dysregulation: on one hand, activation of the Unfolded Protein Response (UPR) and interleukin signalling pathways, reflecting an integrated cellular stress response; on the other hand, significant alterations in cell cycle control pathways, especially ATR activation and the G1/S transition, which were strongly associated with the trisomy groups (Figure 4D and Table S7). Notably, a subset of nine hub genes (e.g., *MCM4*, *CDC7*, *CDC25A*, *DHFR*, *HSPA5*, *DNAJC3*, *CALR*, *HSP90B1* and *SRGN*) exhibited consistent differential expression across trisomy subtypes (Figure 4E). These genes are functionally linked to cell cycle regulation, proteostasis, and inflammatory signalling, reinforcing the notion of shared cellular dysfunctions driven by aneuploidy. In addition, the set of 60 DEGs enriched in Gene Ontology terms and Reactome pathways is presented in Tables S6 and S7.

### 3.8 Drug Sensitivity Analysis Reveals Differential Therapeutic Targets in Trisomies 8, 21 and 13+22

**FIGURE 5:**
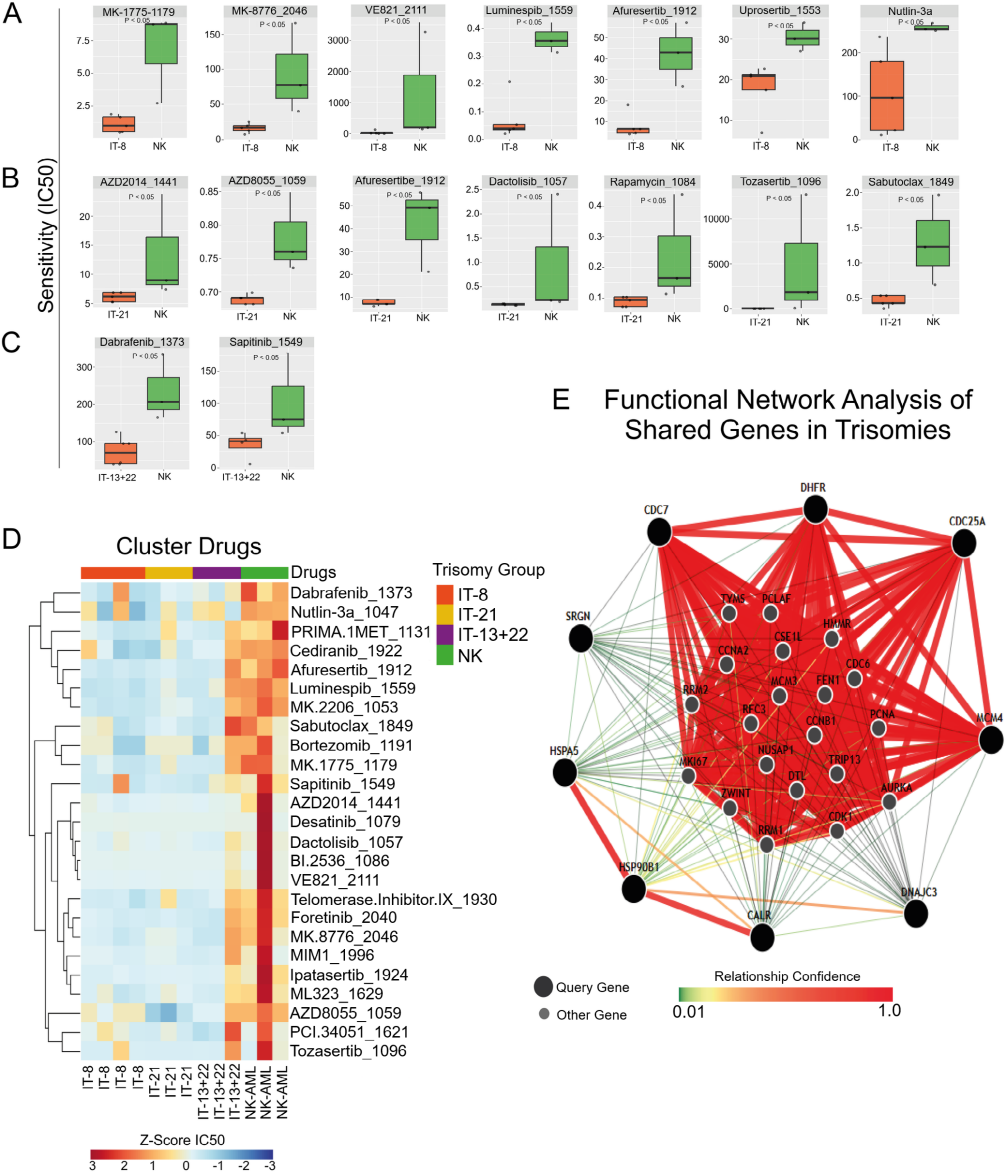
Drug Sensitivity Prediction Based on Gene Expression Profiles in Trisomy Groups. (A–C) Boxplots showing predicted drug sensitivity (IC50 values) for selected compounds in each trisomy group versus cytogenetically normal (NK) patients, based on OncoPredict analysis. (A) Patients with trisomy 8 (IT‐8) showed increased predicted sensitivity to agents targeting cell cycle checkpoints (MK‐1775, MK‐8776), DNA damage response (VE821), the p53 pathway (Nutlin‐3a), PI3K/AKT/mTOR signalling (Afuresertib, AZD8055) and proteostasis (Luminespib). (B) Patients with trisomy 21 (IT‐21) demonstrated heightened predicted sensitivity to mitotic inhibitors (BI.2536, Tozasertib, Dactolisib), apoptotic modulators (Sabutoclax) and PI3K/mTOR inhibitors (AZD2014, Afuresertib, Rapamycin), suggesting dysregulation of proliferative and survival pathways. (C) The IT‐13+22 group exhibited a more limited sensitivity profile, with significant responses to Dabrafenib (ERK/MAPK pathway) and Sapitinib (EGFR/ERBB family inhibitor). (D) Heatmap showing *Z*‐score‐transformed predicted IC50 values for 26 drugs across all trisomy groups. Lower *Z*‐scores (blue) indicate higher predicted drug sensitivity. Hierarchical clustering using Euclidean distance reveals distinct group‐specific drug response signatures. (E) Functional interaction network of the nine genes with the lowest fold changes across isolated trisomies, constructed using GIANT (Genome‐scale Integrated Analysis of gene Networks in Tissues; available at: https://giant.princeton.edu). The bone marrow‐specific dataset was applied to generate an integrated network module, highlighting interactions related to cell cycle regulation, DNA replication and protein homeostasis. Edge thickness and colour indicate the confidence level of predicted functional associations.

The drug sensitivity analysis performed with OncoPredict revealed distinct profiles among the trisomy groups (IT‐8, IT‐21 and IT‐13+22), uncovering potential therapeutic vulnerabilities specific to each subtype (Figure 5A–C). In the IT‐8 group, several compounds demonstrated significantly increased sensitivity compared to controls, particularly drugs targeting cell cycle checkpoints, such as MK‐1775 (a *WEE1* and *PLK1* inhibitor), MK‐8776 (inhibitor of *CHEK1*, *CHEK2* and *CDK2*) and Nutlin‐3a, which targets the p53 pathway. Additional compounds of interest included Luminespib (HSP90 inhibitor), Afuresertib (*AKT* inhibitor) and VE821 (ATR inhibitor), indicating vulnerabilities in DNA damage response and proteostasis mechanisms (Figure 5A). The full list of compounds with differential predicted sensitivity across the trisomy groups, including statistical significance and drug targets, is available in Table S8.

In the IT‐21 group, enhanced sensitivity was observed for several mitotic inhibitors, including BI.2536 (PLK1–3 inhibitor), Tozasertib (AURKA–C inhibitor) and Dactolisib (dual PI3K/mTOR inhibitor). Notably, drugs modulating the PI3K/mTOR axis (e.g., AZD2014, Afuresertib, Rapamycin) and apoptotic regulation (Sabutoclax) also showed altered response in this group (Figure 5B). In the IT‐13+22 group, only two compounds reached statistical significance: Dabrafenib, a selective BRAF inhibitor acting through the ERK/MAPK signalling pathway and Sapitinib, which targets *EGFR*, *ERBB2* and *ERBB3* pathways, suggesting a more restricted but defined drug response profile (Figure 5C).

Hierarchical clustering of drug response *Z*‐scores (Figure 5D) further confirmed these group‐specific patterns, revealing distinct clusters of compounds preferentially active in each trisomy subtype. Finally, protein–protein interaction analysis of the 60 shared DEGs (Figure 5E) highlighted highly connected functional modules, including regulators of cell cycle control (e.g., *MCM4*, *CDC7*, *CDC25A*, *DHFR*), stress response (*HSPA5*, *DNAJC3*, *CALR*, *HSP90B1*), proteostasis (*DNAJC3*, *HSPA5*, *CALR*, *HSP90B1*) and inflammatory signalling (*HSP90B1*), reinforcing the notion of convergent biological vulnerabilities across trisomies.

## 4 Discussion

This study identified ITs in AML patients, without observing significant age differences between the trisomy and normal karyotype groups. No direct association was found between ITs and advanced age. Few significant clinical differences were observed and these did not correlate with overall survival.

Results indicate that although induction therapy and Allo‐SCT were more common among patients, the complete remission rate and median OS did not substantially differ. This suggests that factors beyond clinical characteristics and treatments, such as performance status, comorbidities and genetic factors, may impact survival outcomes [39].

Genomic analysis revealed trisomy 8 as the most frequent, followed by trisomies of chromosomes 13, 14 and 21, in line with previous studies [1, 2]. Key mutations in genes regulating epigenetic pathways, such as *DNMT3A*, *IDH1‐2*, *ASXL1* and *TET2*, were also identified and have been extensively studied in disease pathogenesis, particularly in older patients [40].

Studies in patients with isolated trisomies, such as 8, 13, 14 and 21, have identified molecular alterations in genes related to metabolic pathways, RNA processing and cell signalling, which influence AML development, prognosis, and treatment [1, 2]. Trisomy 8 is commonly associated with alterations in epigenetic genes and is linked to poorer outcomes compared with patients with a normal karyotype, despite its classification as an intermediate‐risk abnormality [3, 6].

Clinically relevant mutations were identified in *DNMT3A* (Arg882His) and *IDH1/2* (Arg132Cys/Arg140Gln) in trisomies 8 and 13, both involved in DNA methylation regulation and metabolite‐driven leukemogenesis [41]. Mutations in *TET2* and *ASXL1* were also detected, further supporting their role in AML pathogenesis. When considering all isolated trisomies together, we observed distinct combinations of driver mutations across cases, involving genes related to epigenetic control, RNA splicing, signalling, and transcriptional regulation—features that characterise the molecular heterogeneity of AML. Analysis of VAF values in individual samples indicated that most mutations exhibited similar allelic burdens, suggesting the predominance of major clonal populations. Nevertheless, the coexistence of multiple somatic driver mutations in some trisomy 8 cases (e.g., *DNMT3A*, *KRAS*, *FLT3*, *NPM1*, *SRSF2*, *ASXL1*) may indicate underlying clonal complexity; however, a more detailed inference of clonal structure would require dedicated analyses beyond the scope of this study.

While direct associations with prognosis are not yet clear, these mutations indicate increased disease complexity. Previous studies have suggested that critical driver mutations often precede large chromosomal alterations, such as trisomies, which may subsequently arise as secondary events that expand existing clones and contribute to genomic instability in AML Defects in mitotic checkpoints contribute to chromosomal instability, a hallmark of hematologic malignancies and are strongly associated with leukaemia progression [42].

RNA‐seq analysis of trisomies 8, 21, and 13–22 revealed gene expression changes that distinguish these groups from normal karyotype (NK) samples. PCA and heat maps showed distinct gene expression patterns, with 2579 DEGs across trisomies. IT‐8 had the largest number (1336 DEGs), consistent with studies linking trisomy 8 to cell cycle and proliferation dysregulation [43].

Although the number of cases per trisomy group was limited, all DEG analyses incorporated dispersion shrinkage and normalisation procedures to minimise sample‐specific effects. The use of three independent algorithms (DESeq2 [44], edgeR [45], and Limma [46]), together with the requirement for concordant DEGs, further reduced method‐specific artefacts and minimised the influence of outliers. Principal component and clustering analyses confirmed that samples grouped primarily by karyotype rather than by individual‐sample variability. Functional enrichment analyses of DEGs using GSEA consistently indicated that the identified transcriptomic signatures reflect transcriptional patterns specific to each trisomy group.

Hallmark enrichment analysis revealed shared and group‐specific transcriptional changes among trisomies. Consistent downregulation of the G2M Checkpoint pathway suggests impaired mitotic control, a feature of chromosomal instability (CI) in leukaemia. Aberrant chromosome segregation may foster a pro‐inflammatory environment and affect leukaemic cell therapy response [47].

Modest activation of *MYC* Targets V1 in IT‐8, in contrast to variable regulation in IT‐13+22, suggests that trisomy‐specific effects and inter‐individual variability influence MYC‐driven proliferation. In IT‐8, concurrent enrichment of stress‐related pathways—Reactive Oxygen Species (ROS), *KRAS* signalling, and the Unfolded Protein Response (UPR)—reflects cellular adaptation to heightened oxidative and proteotoxic stress. These findings are consistent with studies linking trisomy 8 to genomic instability and proteotoxic burden [48]. Aneuploidy‐induced proteotoxic stress, driven by protein overload, triggers compensatory mechanisms such as chaperone upregulation, autophagy and UPR to maintain proteostasis [49].

While gene dosage effects are generally expected in trisomies, transcriptional outcomes can be modulated by compensatory mechanisms, epigenetic regulation, and post‐transcriptional processes [50]. In IT‐8, the modest upregulation of *MYC* targets likely reflects the biological complexity of trisomy 8 rather than a straightforward gene dosage effect. Such variability is particularly plausible in small patient cohorts and is consistent with previous observations in AML and other trisomies, where chromosomal gains do not always lead to proportional increases in gene expression [51].

The IT‐21 group showed a distinct transcriptional profile, highlighted by strong enrichment of the Epithelial‐Mesenchymal Transition (EMT) pathway, suggesting increased phenotypic plasticity and stem cell–like traits such as migration, therapy resistance and disease persistence [52]. Upregulation of Hedgehog and mTORC1 signalling further indicates aberrant activation of developmental and metabolic pathways involved in stem cell self‐renewal and leukemogenesis [53]. Additionally, enrichment of Oxidative Phosphorylation suggests a metabolic shift toward enhanced mitochondrial respiration, consistent with metabolic reprogramming in hematologic malignancies [54].

In contrast, the IT‐13+22 group showed a strong inflammatory signature, with enrichment of Inflammatory Response, *IL6–JAK–STAT3*, *TNF‐alpha/NF‐κB* and Interferon (alpha/gamma) pathways, suggesting dysregulated immunity that may promote immune evasion or chronic inflammation [55]. Enrichment of Apoptosis and Angiogenesis pathways also points to altered cell death and vascular remodelling, potentially impacting leukaemic cell survival and bone marrow interactions [56].

Integrated analysis revealed a shared molecular signature of 60 DEGs consistently altered across all trisomy groups. These genes were enriched in pathways related to chromatin remodelling, cell cycle control and DNA repair, underscoring the role of genomic instability as a central feature of isolated trisomies [57].

Gene Ontology analysis revealed changes in mitotic spindle organisation, focal adhesion and organelle integrity, suggesting a role in chromosomal missegregation. In particular, improper kinetochore‐microtubule attachments—where kinetochores function as dynamic and adaptable signalling hubs regulating mitotic progression—may compromise chromosome alignment and segregation fidelity [58]. Concurrently, the upregulation of genes related to protein binding, ATP metabolism and chaperone function suggests an adaptive response to mitigate proteotoxic stress commonly associated with aneuploidy [59].

Reactome pathway enrichment highlighted two main axes of dysregulation: the Unfolded Protein Response (UPR) and interleukin signalling, reflecting chronic cellular stress [60] and key alterations in cell cycle progression, particularly ATR signalling and G1/S transition checkpoints [61]. These findings reveal convergent mechanisms of dysfunction in aneuploid haematopoiesis, potentially exploitable for therapeutic targeting.

The drug sensitivity analysis using OncoPredict revealed distinct vulnerability profiles across trisomy groups. In IT‐8, increased sensitivity to cell cycle checkpoint inhibitors (e.g., MK‐1775/MK‐8776) [62] and Nutlin‐3a (p53 pathway) suggests a role for checkpoint and genomic instability [63]. Sensitivity to Afuresertib, *VE821* and further points to disrupted DNA damage response and proteostasis mechanisms in this group [64, 65, 66].

In IT‐21, a distinct drug sensitivity profile emerged, with increased responsiveness to mitotic inhibitors (BI‐2536, targeting *PLK1–3*; Tozasertib, targeting *AURKA–C*), PI3K/mTOR inhibitors (AZD2014, Rapamycin) and the apoptosis modulator Sabutoclax [67, 68, 69, 70]. These patterns indicate dysregulation of mitotic progression, survival, and proliferative signalling, suggesting potential therapeutic opportunities for patients with trisomy 21. In contrast, IT‐13+22 displayed a narrower response, with sensitivity limited to Dabrafenib (targeting *ERK*/*MAPK*) and Sapitinib (*EGFR*/*ERBB*), indicating fewer but specific vulnerabilities.

Supporting this, analysis of 60 DEGs shared across all trisomy subgroups revealed 9 hub genes within a Bayesian network based on human bone marrow data. This module was enriched for key processes: cell cycle control (*MCM4*, *CDC7*, *CDC25A*, *DHFR*), proteostasis and stress response (*HSPA5*, *DNAJC3*, *CALR*, *HSP90B1*) and inflammatory signalling (*HSP90B1*). These findings highlight potential targets for trisomy‐specific therapies aimed at cell cycle checkpoints, stress pathways and proliferative signals.

In conclusion, this study demonstrates the molecular heterogeneity of IT, characterised by distinct genomic and transcriptomic alterations in pathways related to the cell cycle, inflammation and metabolism. Integrative analyses revealed recurrent disruptions in epigenetic regulation, proteostasis and drug response, suggesting potential targets for therapy. Despite cohort limitations, these findings offer a solid basis for further validation and therapeutic development.

## Author Contributions

**Jersey Heitor da S. Maués:** conceptualization (equal), data curation (equal), formal analysis (equal), investigation (equal), methodology (equal), resources (equal), software (equal), supervision (equal), validation (equal), visualization (equal), writing – original draft (equal), writing – review and editing (equal). **Bruno Kosa L. Duarte:** conceptualization (equal), formal analysis (equal), investigation (equal), methodology (equal), supervision (equal), writing – original draft (supporting). **Maria Carolina C. M. Svidnicki:** data curation (equal), formal analysis (equal), methodology (equal), writing – original draft (equal). **Herton Luiz A. S. Filho:** methodology (equal), investigation (equal). **Fernanda Soares Niemann:** methodology (equal). **Adriana da Silva S. Duarte:** methodology (equal). **Paula de Melo Campos:** investigation (equal), supervision (equal). **Pedro M. Moraes‐Vieira:** conceptualization (equal), methodology (equal), supervision (equal). **Sara Teresinha Olalla Saad:** conceptualization (equal), formal analysis (equal), funding acquisition (equal), investigation (equal), project administration (equal), supervision (equal), writing – original draft (equal), writing – review and editing (equal).

## Disclosure

Permission to reproduce material from other sources: No previously published figures, tables, or data requiring reproduction permission were used in this manuscript.

## Ethics Statement

All procedures were approved by the Research Ethics Committee of the University of Campinas (UNICAMP), protocol number CAAE 45254321.7.0000.5404—opinion 5056809.

## Consent

Written informed consent was obtained from all patients or their legal guardians prior to study inclusion, in accordance with the Declaration of Helsinki principles.

## Conflicts of Interest

The authors declare no conflicts of interest.

## Supporting information


**Table S1:** Clinical and Cytogenetic Characteristics of AML Patients with Isolated Trisomies (IT) and Normal Karyotype (NK).
**Table S2:** Composition of the NGS Panel of genes used in this study.
**Table S3:** Genomic results of NK‐AML patients.
**Table S4:** Summary of sequencing quality metrics and read mapping statistics.
**Table S5:** Gene set enrichment analysis.
**Table S5A:** (IT‐8): Hallmark gene set enrichment analysis in IT‐8 trisomy group. NES, *p*‐values and FDRs are reported.
**Table S5B:** (IT‐21): Hallmark gene set enrichment analysis in IT‐21 trisomy group.
**Table S5C:** (IT‐13+22): Hallmark gene set enrichment analysis in IT‐13+22 trisomy group.
**Table S6:** List of 60 DEGs commonly identified across the three trisomy groups (IT‐8, IT‐21 and IT‐13+22).
**Table S7:** Results of Gene Ontology (GO) enrichment analysis for differentially expressed genes, highlighting the most significant biological processes, molecular functions and cellular components associated with the data.
**Table S8:** Enriched Reactome pathways (terms) among the 60 DEGs. Columns include: the Reactome identifier and pathway name (Term), number and percentage of DEGs involved (Count, %), unadjusted *p*‐value (*p*Value), the list of contributing genes (Genes), and the adjusted *p*‐value using False Discovery Rate (FDR).
**Table S9:** Drug sensitivity analysis reveals differential therapeutic vulnerabilities using OncoPredict.
**Figure S1:** Main stages of analysis and processing of genomic data generated with NGS: (1) Quality control of raw data, (2) sequence alignment, variant calling, and annotation in VCF files, (3) annotation and filtering of genetic variants using BCF tools + (Processing and filtering in R), and graphical visualisation with IGV for variant identification and classification.
**Figure S2:** Overall survival analysis between the NK and IT groups of adult patients with acute myeloid leukaemia treated with intensive therapy. OS analysis of 71 patients in our cohort, including 57 in the NK group and 14 in the IT group. The log‐rank test was used to differentiate the groups.

## Data Availability

The gene expression data supporting the findings of this study will soon be available in the Sequence Read Archive (SRA) under the accession number PRJNA1233239. The datasets analyzed in this study are available from the corresponding author upon reasonable request.
