# Supplementary material for: Integrated Genomic and Transcriptomic Profiling of Isolated Trisomies in AML Reveals Cell Cycle Dysregulation and Therapeutic Vulnerabilities
Source: J Cell Mol Med. 2025 Nov 27;29(22):e70941. doi: 10.1111/jcmm.70941 (PMC12660057; doi:10.1111/jcmm.70941)
Supplement: Supplementary file 1 — Table S1: Clinical and Cytogenetic Characteristics of AML Patients with Isolated Trisomies (IT) and Normal Karyotype (NK). Table S2: Composition of the NGS Panel of genes used in this study. Table S3: Genomic results of NK‐AML patients. Table S4: Summary of sequencing quality metrics and read mapping statistics. Table S5: Gene set enrichment analysis. Table S5A: (IT‐8): Hallmark gene set enrichment analysis in IT‐8 trisomy group. NES, p‐values and FDRs are reported. Table S5B: (IT‐21): Hallmark gene set enrichment analysis in IT‐21 trisomy group. Table S5C: (IT‐13+22): Hallmark gene set enrichment analysis in IT‐13+22 trisomy group. Table S6: List of 60 DEGs commonly identified across the three trisomy groups (IT‐8, IT‐21 and IT‐13+22). Table S7: Results of Gene Ontology (GO) enrichment analysis for differentially expressed genes, highlighting the most significant biological processes, molecular functions and cellular components associated with the data. Table S8: Enriched Reactome pathways (terms) among the 60 DEGs. Columns include: the Reactome identifier and pathway name (Term), number and percentage of DEGs involved (Count, %), unadjusted p‐value (pValue), the list of contributing genes (Genes), and the adjusted p‐value using False Discovery Rate (FDR). Table S9: Drug sensitivity analysis reveals differential therapeutic vulnerabilities using OncoPredict. Figure S1: Main stages of analysis and processing of genomic data generated with NGS: (1) Quality control of raw data, (2) sequence alignment, variant calling, and annotation in VCF files, (3) annotation and filtering of genetic variants using BCF tools + (Processing and filtering in R), and graphical visualisation with IGV for variant identification and classification. Figure S2: Overall survival analysis between the NK and IT groups of adult patients with acute myeloid leukaemia treated with intensive therapy. OS analysis of 71 patients in our cohort, including 57 in the NK group and 14 in the IT group. The l [file JCMM-29-e70941-s001.docx]

**Supplementary Information**

**Manuscript Title:**

**Integrated Genomic and Transcriptomic Profiling of Isolated Trisomies in AML Reveals Cell Cycle Dysregulation and Therapeutic Vulnerabilities**

Authors:
Jersey Heitor da S. Maués¹, Bruno Kosa L. Duarte¹, Maria Carolina C. M. Svidnicki¹, Herton Luiz A. S. Filho¹, Fernanda Soares Niemann¹, Adriana da Silva S. Duarte¹, Paula de Melo Campos¹, Pedro M. Moraes-Vieira², Sara Teresinha Olalla Saad¹

Affiliations:

1. National Institute of Science and Technology of Blood (INCT) and Hematology and Transfusion Medicine Center (Hemocentro), University of Campinas, Campinas, SP, 13083-970, Brazil
2. Laboratory of Immunometabolism, Department of Genetics, Evolution, Microbiology and Immunology– Institute of Biology, University of Campinas (UNICAMP), Campinas, SP 13083-862, Brazil

**Corresponding Author:**

Correspondence to: jhmaues@unicamp.br and sara@unicamp.br

Rua Carlos Chagas, 480, Cidade Universitária, Campinas, SP, Brazil

Zip code: 13083-878

Phone number: +55 19 35218740

**Supplementary Table 1: Clinical and Cytogenetic Characteristics of AML Patients with Isolated Trisomies (IT) and Normal Karyotype (NK).**

| **Patients** | **Dx** | **FAB** | **Age** | **Karyotype** | **Trisomies** | **% blasts in BM** |
| --- | --- | --- | --- | --- | --- | --- |
| 1 | LMA | M2 | 54 | 47, XY, +8 | Trissomia +8 | 54% |
| 2 | LMA | M4 | 47 | 47, XX, +8[08]/46, XX[12] | Trissomia +8 | 75% |
| 3 | LMA | M4 | 47 | 47,XY,+8[10]/46,XY[10] | Trissomia +8 | 84% |
| 4 | LMA | M6 | 64 | 47, XY, +8 | Trissomia +8 | 27.50% |
| 5 | LMA | M1 | 29 | 47, XX, +8[20] | Trissomia +8 | 86% |
| 6 | LMA | M1 | 49 | 47, XX, +21[20] | Trissomia +21 | 72% |
| 7 | LMA | M1 | 49 | 47, XX, +21[20] | Trissomia +21 | 72% |
| 8 | LMA | M4 | 31 | 47,XX,+21[30] | Trissomia +21 | 94.65% |
| 9 | LMA | M1 | 61 | 47,XY,+13[08]/46,XY[12] | Trissomia +13 | 89% |
| 10 | LMA | M1 | 70 | 47,XX,+13[04]/46[16] | Trissomia +13 | 91.60% |
| 11 | LMA | M4 | 21 | 46, XY, +22[24]; 46, XY [01] | Trissomia +22 | 84% |
| 12 | LMA | M4 | 21 | 46, XY, +22[24]; 46, XY [01] | Trissomia +22 | 84% |
| 13 | LMA | M2 | 59 | 46,XX[20] | NK | 65% |
| 14 | LMA | M2 | 76 | 46,XX[20] | NK | 59% |
| 15 | LMA | M2 | 73 | 46,XX[20] | NK | 64% |

**Supplementary Table 2: Composition of the NGS Panel of genes used in this study.**

| **Method** | **Instrument** | **Content Specifications** | **Target Genes** | **Panel/Library Prep.** |
| --- | --- | --- | --- | --- |
| NGS, targeted sequencing | MiSeq System | All exons | *ASXL1, BCOR, CALR, CEBPA, ETV6, EZH2, IKZF1, NF1, PHF6, PRPF8, RB1, RUNX1, SH2B3, STAG2, TET2, TP53, ZRSR2* | AmpliSeq™ myeloid panel from Illumina® DNA panel  AmpliSeq™ Library PLUS for Illumina® |
|  | MiSeq System | Hotspot mutations | *ABL, BRAF, CBL, CSF3R, DNMT3A, FLT3, GATA2, HRAS, IDH1, IDH2, JAK2, KIT, KRAS, MPL, MYD88, NPM1, NRAS, PTPN11, SETBP1, SF3B1, SRSF2, U2AF1 and WT1* |  |

**Supplementary Table 3: Genomic results of NK-AML patients**

| **Paciente** | **Genes** | **Chr** | **Classification** | **txChange** | **aaChange** | **Exons** | **VAF** | **Confidence** | **Depth** | **Region** | **Clinvar** | **Aggregated Frequency** |
| --- | --- | --- | --- | --- | --- | --- | --- | --- | --- | --- | --- | --- |
| **1** | ***TP53*** | 17 | Frame_Shift_Del | c.721del | Ser241Profs*6 | 7 | 0.57 | HIGH | 276 | Exonic | Pathogenic | N/A |
|  | ***KIT*** | 4 | Missense_Mutation | c.2447A>T | Asp816Val | 17 | 0.20 | MEDIUM | 555 | Exonic | Likely Pathogenic | 0.0000 |
|  |  |  |  |  |  |  |  |  |  |  |  |  |
| **2** | ***TP53*** | 17 | Missense_Mutation | c.403T>C | Cys135Arg | 5 | 0.63 | HIGH | 610 | Exonic | Pathogenic | N/A |
|  | ***CEBPA*** | 19 | Frame_Shift_Del | c.158del | Gly53Alafs*107 | 1 | 0.08 | MEDIUM | 89 | Exonic | Likely Pathogenic | N/A |
| **3** | ***TP53*** | 17 | Splice_Site | c.559+1G>A |  | 5 | 0.43 | MEDIUM | 1131 | Splice Donor | Pathogenic | 0.0000 |
|  | ***TP53*** | 17 | Missense_Mutation | c.584T>C | Ile195Thr | 6 | 0.40 | MEDIUM | 555 | Exonic | Pathogenic | <0.01 |
|  | ***IKZF1*** | 7 | Missense_Mutation | c.476A>G | Asn159Ser | 5 | 0.35 | MEDIUM | 692 | Exonic | Pathogenic | <0.01 |
|  | ***TET2*** | 4 | Missense_Mutation | c.356A>G | Asn119Ser | 3 | 0.47 | HIGH | 577 | Exonic | Uncertain | <0.01 |
| **4** | ***NRAS*** | 1 | Missense_Mutation | c.181C>A | Gln61Lys | 3 | 0.47 | HIGH | 785 | Exonic | Pathogenic | 0.0000 |
|  | ***EZH2*** | 7 | Missense_Mutation | c.1753T>G | Cys585Gly | 15 | 0.49 | HIGH | 599 | Exonic | Likely Pathogenic | N/A |
|  | ***CEBPA*** | 19 | Frame_Shift_Ins | c.155_156insCTTCC | Gly53Phefs*109 | 1 | 0.06 | MEDIUM | 159 | Exonic | Likely Pathogenic | N/A |
| **5** | ***TP53*** | 17 | Missense_Mutation | c.488A>G | Tyr163Cys | 5 | 0.09 | MEDIUM | 2212 | Exonic | Pathogenic | <0.01 |
|  | ***TP53*** | 17 | Missense_Mutation | c.535C>T | His179Tyr | 5 | 0.10 | MEDIUM | 2002 | Exonic | Pathogenic | N/A |
| **6** | ***TP53*** | 17 | Missense_Mutation | c.1010G>A | Arg337His | 10 | 0.34 | MEDIUM | 450 | Exonic | Pathogenic | <0.01 |
|  | ***TP53*** | 17 |  | c.852dup | Glu285Argfs*21 | 8 | 0.19 | MEDIUM | 449 | Exonic | Likely Pathogenic | N/A |

N/A: Not Applicable

**Supplementary Table 4: Summary of sequencing quality metrics and read mapping statistics.**

| **Patients** | **Total_Reads** | **% Aligned Reads** | **% Multi-Mapped** | **% Duplicates** | **Mean Fragment Length (bp)** | **% Reads Aligned to Coding Exons** | **% Mito. reads** | **% Ribo. reads**. | **% Coding_Exons** | **% UTR** | **% Introns** | **% Intergenic** | **Genes 10X** |
| --- | --- | --- | --- | --- | --- | --- | --- | --- | --- | --- | --- | --- | --- |
| 1 | 52071202 | 94.79% | 5.85% | 0.40% | 172.9 | 50.94% | 8.49 | 3.59 | 50.97 | 33.02 | 13.23 | 2.78 | 11902 |
| 2 | 52409358 | 91.46% | 6.05% | 0.41% | 174.53 | 52.61% | 14.29 | 1.90 | 52.55 | 31.32 | 12.56 | 3.57 | 11216 |
| 3 | 53831897 | **96.45%** | 4.99% | 0.32% | 176.86 | 53.62% | 17.99 | 3.33 | 53.37 | 31.34 | 12.39 | 2.91 | 10685 |
| 4 | 55154784 | 96.09% | **13.74%** | 4.41% | 166.98 | **57.42%** | 10.60 | 1.96 | 57.38 | 28.85 | 10.08 | 3.7 | 11856 |
| 5 | 64445483 | 96.12% | 5.95% | 0.46% | 172.41 | 55.55% | 8.83 | 2.00 | 55.46 | 30.27 | 11.62 | 2.65 | 11310 |
| 6 | 62890151 | 95.78% | 4.88% | 0.29% | **179.61** | 48.82% | 10.57 | 1.68 | 48.46 | 31.61 | 16.59 | 3.34 | 11771 |
| 7 | 36967239 | **89.65%** | 4.53% | 0.22% | 176.07 | 47.42% | 9.19 | 1.57 | 47.44 | 32.72 | 16.28 | 3.55 | 11133 |
| 8 | 68279616 | 94.74% | 5.28% | 0.23% | 182.11 | 43.62% | 18.07 | 2.04 | 43.64 | 30.07 | 20.77 | 5.52 | 12176 |
| 9 | 69890095 | 96.07% | 4.40% | 0.12% | 181.04 | 40.61% | 11.08 | 1.60 | 40.70 | 30.93 | 23.31 | 5.07 | 12653 |
| 10 | 41390915 | 93.89% | 4.75% | 0.16% | 171.18 | 40.53% | 14.80 | 1.74 | 40.43 | 28.08 | 26.46 | 5.03 | 11170 |
| 11 | 51426335 | 93.48% | 3.24% | 0.12% | 187.47 | 43.52% | 12.84 | 2.80 | 43.37 | 32.91 | 20.15 | 3.58 | 11377 |
| 12 | 48646081 | 95.21% | 3.32% | 0.14% | 177.08 | **42.45%** | 13.84 | 1.52 | 42.43 | 33.16 | 20.7 | 3.71 | 11360 |
| 13 | 50834043 | 94.63% | 4.35% | 0.15% | 179.27 | 43.68% | 19.64 | 1.52 | 43.84 | 32.2 | 19.97 | 3.99 | 11432 |
| 14 | 75050137 | 94.05% | 5.09% | 0.19% | 173.48 | 43.21% | 13.47 | 1.11 | 43.22 | 32.33 | 18.92 | 5.53 | 12803 |
| 15 | 84542514 | 94.78% | 3.87% | 0.29% | 171.41 | 42.92% | 13.28 | 1.23 | 43.04 | 33.52 | 19.81 | 3.62 | 12610 |

**Explanation of columns:**

- Total Reads: Total number of sequenced reads per sample (ranging from ~37M to 85M).
- % Aligned Reads: Percentage of reads aligned to the reference genome (sample3 had the highest rate at 96.45%, while sample7 showed the lowest at 89.65%).
- % Multi-Mapped: Fraction of reads aligning to multiple loci (sample4 showed the highest value at 13.74%, which may reflect mapping in repetitive regions).
- % Duplicates: Percentage of duplicated fragments (sample4 had the highest duplication rate at 4.41%, whereas most other samples remained below 0.5%).
- Mean Fragment Length (bp): Average insert size across reads (sample11 showed the longest fragments at 187.47 bp, while sample4 had the shortest at 166.98 bp).
- % Reads Aligned to Coding Exons: Fraction of reads mapping to coding regions (sample4 showed the highest at 57.42%, while sample10 had the lowest at 40.53%).
- % Mitochondrial Reads: Proportion of reads aligning to mitochondrial genes (ranging from 8.49% in sample1 to 19.64% in sample13).
- % Ribosomal Reads: Proportion of reads aligning to ribosomal RNA (lowest at 1.11% in sample14 and highest at 3.59% in sample1).
- Genomic Distribution of Reads: Distribution of mapped reads across genomic features – coding exons, untranslated regions (UTRs), introns, and intergenic sequences. For example, sample4 had the highest representation in coding exons (57.38%), while sample9 showed the largest fraction in intronic regions (23.31%).
- Genes 10X: Number of genes detected with a minimum of 10 reads, representing a balance between sensitivity and robustness of gene detection (ranging from 10,685 in sample3 to 12,803 in sample14).

**Supplementary Table 5: Gene set enrichment analysis**

**Supplementary Table S5A (IT-8): Hallmark gene set enrichment analysis in IT-8 trisomy group. NES, p-values and FDRs are reported.**

| NAME | SIZE | ES | NES | NOM P-val | FDR q-val | FWER P-val |
| --- | --- | --- | --- | --- | --- | --- |
| HALLMARK_MYC_TARGETS_V1 | 196 | 0.250 | 1.044 | 0.000 | 0.240 | 1.000 |
| HALLMARK_OXIDATIVE_PHOSPHORYLATION | 178 | 0.428 | 0.020 | 0.000 | 0.000 | 0.000 |
| HALLMARK_INTERFERON_GAMMA_RESPONSE | 139 | 0.320 | 0.143 | 0.000 | 0.068 | 0.300 |
| HALLMARK_DNA_REPAIR | 124 | 0.335 | 0.166 | 0.000 | 0.000 | 0.000 |
| HALLMARK_IL2_STAT5_SIGNALING | 111 | 0.250 | 1.066 | 0.000 | 0.580 | 1.000 |
| HALLMARK_GLYCOLYSIS | 110 | 0.279 | 1.200 | 0.000 | 0.248 | 1.000 |
| HALLMARK_HYPOXIA | 109 | 0.293 | 1.271 | 0.000 | 0.168 | 0.800 |
| HALLMARK_INTERFERON_ALPHA_RESPONSE | 72 | 0.332 | 0.135 | 0.000 | 0.080 | 0.300 |
| HALLMARK_IL6_JAK_STAT3_SIGNALING | 52 | 0.358 | 1.328 | 0.000 | 0.084 | 0.300 |
| HALLMARK_MYC_TARGETS_V2 | 52 | 0.325 | 1.222 | 0.000 | 0.205 | 1.000 |
| HALLMARK_REACTIVE_OXYGEN_SPECIES_PATHWAY | 45 | 0.349 | 1.512 | 0.000 | 0.048 | 0.200 |
| HALLMARK_KRAS_SIGNALING_DN | 34 | 0.408 | 1.388 | 0.000 | 0.060 | 0.300 |
| HALLMARK_E2F_TARGETS | 153 | -0.369 | -1.465 | 0.000 | 0.026 | 0.200 |
| HALLMARK_G2M_CHECKPOINT | 146 | -0.354 | -1.637 | 0.000 | 0.020 | 0.100 |
| HALLMARK_MITOTIC_SPINDLE | 143 | -0.290 | -1.368 | 0.000 | 0.045 | 0.400 |
| HALLMARK_UNFOLDED_PROTEIN_RESPONSE | 94 | -0.265 | -1.274 | 0.000 | 0.111 | 0.600 |
| HALLMARK_KRAS_SIGNALING_UP | 81 | -0.304 | -0.129 | 0.000 | 0.127 | 0.600 |
| HALLMARK_WNT_BETA_CATENIN_SIGNALING | 21 | -0.432 | -1.988 | 0.000 | 0.000 | 0.000 |

**Supplementary Table S5B (IT-21): Hallmark gene set enrichment analysis in IT-21 trisomy group.**

| NAME | SIZE | ES | NES | NOM P-val | FDR q-val | FWER P-val |
| --- | --- | --- | --- | --- | --- | --- |
| HALLMARK_EPITHELIAL_MESENCHYMAL_TRANSITION | 75 | 0.330 | 1.345 | 0.000 | 0.207 | 0.500 |
| HALLMARK_MYC_TARGETS_V2 | 56 | 0.381 | 1.269 | 0.000 | 0.212 | 0.700 |
| HALLMARK_NOTCH_SIGNALING | 21 | 0.499 | 1.312 | 0.000 | 0.206 | 0.600 |
| HALLMARK_HEDGEHOG_SIGNALING | 12 | 0.545 | 1.411 | 0.000 | 0.228 | 0.400 |
| HALLMARK_OXIDATIVE_PHOSPHORYLATION | 192 | -0.280 | -1.317 | 0.000 | 0.129 | 0.900 |
| HALLMARK_G2M_CHECKPOINT | 174 | -0.323 | -1.410 | 0.000 | 0.022 | 0.500 |
| HALLMARK_HEME_METABOLISM | 167 | -0.414 | -2.052 | 0.000 | 0.000 | 0.000 |
| HALLMARK_INTERFERON_GAMMA_RESPONSE | 155 | -0.313 | -1.443 | 0.000 | 0.019 | 0.500 |
| HALLMARK_KRAS_SIGNALING_UP | 98 | -0.338 | -1.436 | 0.000 | 0.019 | 0.500 |
| HALLMARK_PI3K_AKT_MTOR_SIGNALING | 85 | -0.399 | -1.542 | 0.000 | 0.069 | 0.300 |
| HALLMARK_INTERFERON_ALPHA_RESPONSE | 79 | -0.340 | -1.340 | 0.000 | 0.121 | 0.900 |
| HALLMARK_IL6_JAK_STAT3_SIGNALING | 58 | -0.394 | -1.480 | 0.000 | 0.058 | 0.300 |

**Supplementary Table S5C (IT-13+22): Hallmark gene set enrichment analysis in IT-13+22 trisomy group.**

| NAME | SIZE | ES | NES | NOM P-val | FDR q-val | FWER P-val |
| --- | --- | --- | --- | --- | --- | --- |
| HALLMARK_INFLAMMATORY_RESPONSE | 91 | 0.479 | 2.081 | 0.000 | 0.000 | 0.000 |
| HALLMARK_IL6_JAK_STAT3_SIGNALING | 50 | 0.551 | 1.885 | 0.000 | 0.000 | 0.000 |
| HALLMARK_INTERFERON_GAMMA_RESPONSE | 138 | 0.478 | 1.869 | 0.000 | 0.000 | 0.000 |
| HALLMARK_INTERFERON_ALPHA_RESPONSE | 67 | 0.511 | 1.638 | 0.000 | 0.000 | 0.000 |
| HALLMARK_APICAL_JUNCTION | 95 | 0.383 | 1.573 | 0.000 | 0.021 | 0.100 |
| HALLMARK_APICAL_SURFACE | 15 | 0.616 | 1.570 | 0.000 | 0.018 | 0.100 |
| HALLMARK_MITOTIC_SPINDLE | 143 | 0.344 | 1.547 | 0.000 | 0.016 | 0.100 |
| HALLMARK_ANGIOGENESIS | 12 | 0.583 | 1.521 | 0.000 | 0.027 | 0.200 |
| HALLMARK_APOPTOSIS | 107 | 0.352 | 1.500 | 0.000 | 0.047 | 0.300 |
| HALLMARK_IL2_STAT5_SIGNALING | 107 | 0.378 | 1.460 | 0.000 | 0.054 | 0.300 |
| HALLMARK_P53_PATHWAY | 128 | 0.296 | 1.383 | 0.000 | 0.058 | 0.400 |
| HALLMARK_E2F_TARGETS | 159 | -0.436 | -0.217 | 0.000 | 0.000 | 0.000 |
| HALLMARK_MYC_TARGETS_V2 | 45 | -0.519 | -2.155 | 0.000 | 0.000 | 0.000 |
| HALLMARK_MYC_TARGETS_V1 | 193 | -0.399 | -1.985 | 0.000 | 0.000 | 0.000 |
| HALLMARK_UNFOLDED_PROTEIN_RESPONSE | 92 | -0.390 | -1.709 | 0.000 | 0.000 | 0.000 |
| HALLMARK_G2M_CHECKPOINT | 153 | -0.362 | -1.524 | 0.000 | 0.035 | 0.300 |
| HALLMARK_MTORC1_SIGNALING | 166 | -0.215 | -1.151 | 0.000 | 0.446 | 0.900 |

The table presents the results of gene set enrichment analysis (GSEA), including the number of genes in each set (SIZE), the enrichment score (ES) and the normalized enrichment score (NES) adjusted for gene set size. Statistical significance is provided as the nominal p-value (NOM P-val), the false discovery rate (FDR q-val) and the family-wise error rate (FWER P-val).

**Supplementary Table 6: List of 60 DEGs commonly identified across the three trisomy groups (IT-8, IT-21 and IT-13+22).**

| *CHD7, PDE8A, UEVLD, TMEM170B, MPO, CDCA5, MS4A3, ELANE, LNPK, CFH, TLR4, HGF, GOLGA8A, TROAP, DNAJC3, ABCA13, NUSAP1, PKD2, SRGN, F13A1, DSTYK, PRKCI, CDC25A, LIN7A, SLC25A13, IQGAP1, HMGB2, RAD18, B3GNTL1, QSOX2, FTO, DHFR, CDC7, CTSG, HSP90B1, BRD7, RNASE2, MCM4, HSPA5, BPTF, AP3S1, RPL36A-HNRNPH2, FAM107B, FKBP5, ATAD2, AP5B1, CALR, PLD3, PPM1F, LAT2, JUP, PFKL, SIPA1, FSCN1, LY6E, DDAH2, EGFL7, TTC31, PTP4A3, TRIM58* |
| --- |


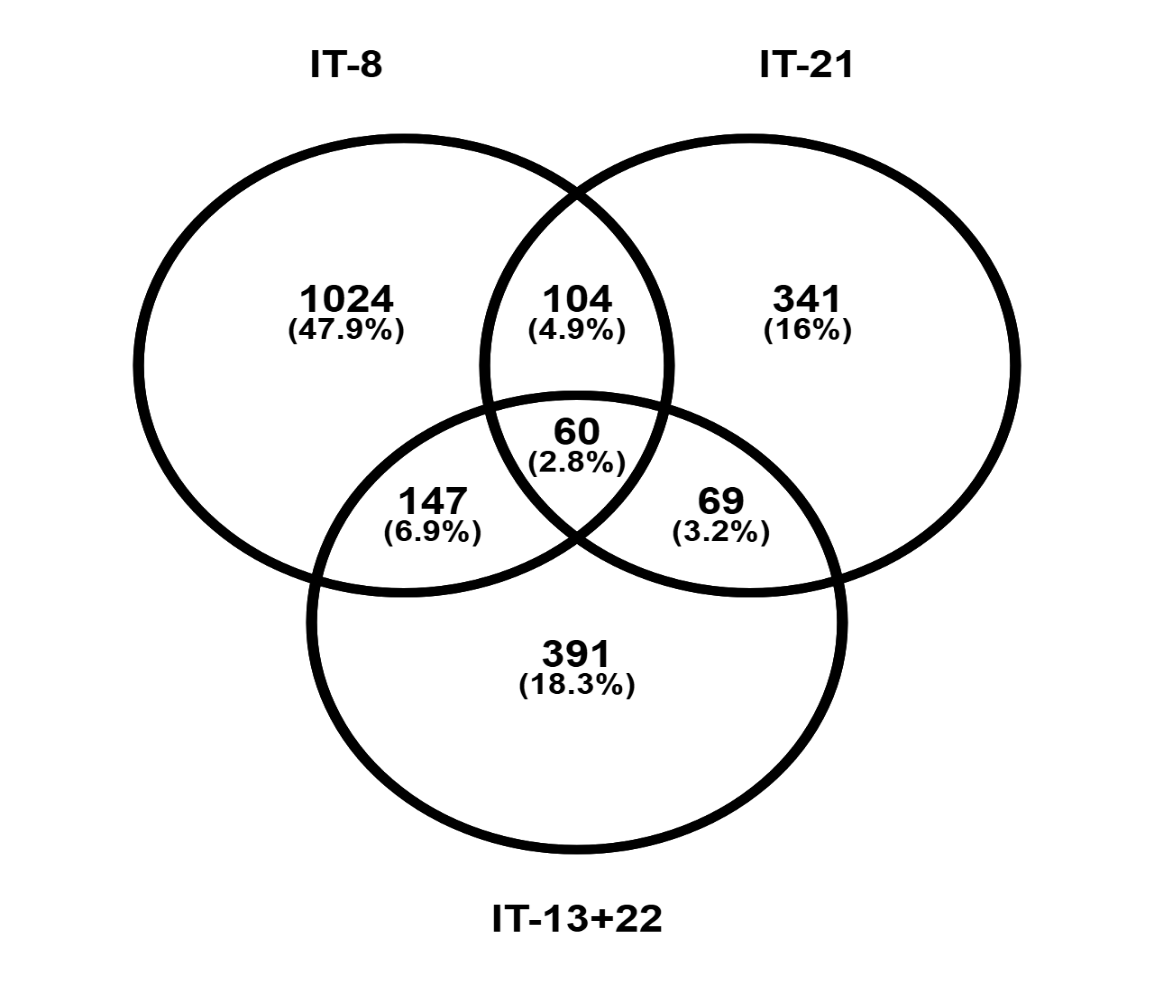


**Supplementary Table 7: Results of Gene Ontology (GO) enrichment analysis for differentially expressed genes, highlighting the most significant biological processes, molecular functions and cellular components associated with the data.**

| **Category** | **Term** | **Count** | **PValue** | **Log10PValue** | **Genes** | **FDR** |
| --- | --- | --- | --- | --- | --- | --- |
| **Biological Process** | GO:0008152-metabolic process | 36 | 0.0040 | 2.397 | *FTO, CFH, CDCA5, CHD7, HMGB2, F13A1, MPO, B3GNTL1, PLD3, PPM1F, HSP90B1, CTSG, QSOX2, BRD7, PDE8A, ELANE, SRGN, PRKCI,*  *HSPA5, RPL36A-HNRNPH2, HGF, ATAD2, CDC7, RNASE2, DNAJC3, DHFR, UEVLD, PFKL, DDAH2, MCM4, TRIM58, CALR, TLR4, RAD18, FKBP5, SLC25A13* | 0.230 |
| **Biological Process** | GO:0006338-chromatin remodeling | 12 | 0.0013 | 2.897 | *DSTYK, PTP4A3, HSPA5, CHD7, ATAD2, HMGB2, MCM4, CDC7, BRD7, CDC25A, PPM1F, BPTF* | 0.145 |
| **Biological Process** | GO:0006508-proteolysis | 10 | 0.0133 | 1.876 | *SRGN, DNAJC3, CFH, HSPA5, HGF, TRIM58, CTSG, CALR, ELANE, HSP90B1* | 0.416 |
| **Biological Process** | GO:0051726-regulation of cell cycle | 10 | 0.0056 | 2.251 | *CDCA5, NUSAP1, CDC7, IQGAP1, CALR, BRD7, PKD2, CDC25A, RAD18, SIPA1* | 0.294 |
| **Biological Process** | GO:0016477-cell migration | 9 | 0.0058 | 2.233 | *PRKCI, PTP4A3, JUP, HGF, FSCN1, HMGB2, CTSG, IQGAP1, ELANE* | 0.294 |
| **Biological Process** | GO:0007010-cytoskeleton organization | 9 | 0.0374 | 1.427 | *PRKCI, FSCN1, NUSAP1, IQGAP1, CALR, PKD2, GOLGA8A, SIPA1, HSP90B1* | 0.550 |
| **Biological Process** | GO:0007049-cell cycle | 9 | 0.0091 | 2.042 | *HGF, CDCA5, NUSAP1, MCM4, CDC7, IQGAP1, PKD2, CDC25A, GOLGA8A* | 0.348 |
| **Biological Process** | GO:0000278-mitotic cell cycle | 7 | 0.0096 | 2.016 | *HGF, CDCA5, NUSAP1, MCM4, CDC7, IQGAP1, CDC25A* | 0.361 |
| **Biological Process** | GO:0006281-DNA repair | 6 | 0.0206 | 1.687 | *FTO, CDCA5, HMGB2, MCM4, CDC7, RAD18* | 0.431 |
| **Biological Process** | GO:1903047-mitotic cell cycle process | 6 | 0.0204 | 1.690 | *CDCA5, NUSAP1, MCM4, CDC7, IQGAP1, CDC25A* | 0.431 |
| **Biological Process** | GO:0043549-regulation of kinase activity | 6 | 0.0086 | 2.065 | *DSTYK, IQGAP1, TLR4, CDC25A, ELANE, PPM1F* | 0.342 |
| **Biological Process** | GO:0045787-positive regulation of cell cycle | 6 | 0.0033 | 2.477 | *CDCA5, NUSAP1, CDC7, CALR, CDC25A, RAD18* | 0.225 |
| **Biological Process** | GO:1901987-regulation of cell cycle phase transition | 5 | 0.0525 | 1.280 | *CDCA5, CDC7, BRD7, PKD2, CDC25A* | 0.655 |
| **Biological Process** | GO:1901990-regulation of mitotic cell cycle phase transition | 5 | 0.0233 | 1.632 | *CDCA5, CDC7, BRD7, PKD2, CDC25A* | 0.452 |
| **Biological Process** | GO:0007076-mitotic chromosome condensation | 2 | 0.0054 | 2.271 | *CDCA5, NUSAP1* | 0.655 |
| **Cellular Component** | GO:0043226-organelle | 52 | 0.0049 | 2.309 | *MS4A3, CHD7, HMGB2, F13A1, PKD2, MPO, ABCA13, NUSAP1, CTSG, AP3S1, QSOX2, PDE8A, SRGN, PRKCI, RPL36A-HNRNPH2, HGF, RNASE2,*  *AP5B1, CDC25A, LAT2, DNAJC3, PTP4A3, DDAH2, FSCN1, TRIM58, MCM4, TLR4, GOLGA8A, SLC25A13, FKBP5, FTO, CFH, CDCA5, LIN7A, IQGAP1,*  *PPM1F, PLD3, HSP90B1, BRD7, ELANE, LNPK, BPTF, JUP, HSPA5, ATAD2, CDC7, DHFR, UEVLD, PFKL, CALR, RAD18, SIPA1* | 0.039 |
| **Cellular Component** | GO:0043229-intracellular organelle | 49 | 0.0117 | 1.930 | *MS4A3, CHD7, HMGB2, F13A1, PKD2, MPO, ABCA13, NUSAP1, CTSG, AP3S1, QSOX2, PDE8A, SRGN, PRKCI, RPL36A-HNRNPH2, HGF, RNASE2, AP5B1,*  *CDC25A, DNAJC3, PTP4A3, DDAH2, FSCN1, TRIM58, MCM4, TLR4, GOLGA8A, SLC25A13, FKBP5, FTO, CDCA5, IQGAP1, PPM1F, PLD3, HSP90B1, BRD7,*  *ELANE, LNPK, BPTF, JUP, HSPA5, ATAD2, CDC7, DHFR, UEVLD, PFKL, CALR, RAD18, SIPA1* | 0.089 |
| **Cellular Component** | GO:0043231-intracellular membrane-bounded organelle | 48 | 0.0024 | 2.625 | *FTO, MS4A3, CDCA5, CHD7, HMGB2, F13A1, IQGAP1, PKD2, MPO, ABCA13, PLD3, PPM1F, HSP90B1, NUSAP1, CTSG, AP3S1, QSOX2, BRD7, PDE8A, ELANE,*  *LNPK, BPTF, SRGN, PRKCI, JUP, HSPA5, RPL36A-HNRNPH2, HGF, ATAD2, CDC7, RNASE2, AP5B1, CDC25A, DNAJC3, DHFR, UEVLD, PTP4A3, PFKL, DDAH2,*  *MCM4, TRIM58, CALR, TLR4, RAD18, SIPA1, GOLGA8A, FKBP5, SLC25A13* | 0.020 |
| **Cellular Component** | GO:0071944-cell periphery | 27 | 0.0261 | 1.583 | *FTO, MS4A3, F13A1, LIN7A, IQGAP1, PKD2, ABCA13, HSP90B1, DSTYK, CTSG, QSOX2, ELANE, SRGN, PRKCI, EGFL7, JUP, HSPA5, RPL36A-HNRNPH2,*  *TMEM170B, LAT2, PTP4A3, FSCN1, CALR, TLR4, SIPA1, LY6E, SLC25A13* | 0.167 |
| **Cellular Component** | GO:0005829-cytosol | 24 | 0.0231 | 1.636 | *FTO, PRKCI, HSPA5, JUP, RPL36A-HNRNPH2, CDCA5, IQGAP1, PKD2, CDC25A, PPM1F, HSP90B1, DNAJC3, DHFR, PFKL, FSCN1, TRIM58, CTSG,*  *CALR, BRD7, PDE8A, ELANE, GOLGA8A, SIPA1, FKBP5* | 0.162 |
| **Cellular Component** | GO:0005911-cell-cell junction | 6 | 0.0168 | 1.774 | *PRKCI, JUP, FSCN1, LIN7A, IQGAP1, PKD2* | 0.121 |
| **Cellular Component** | GO:0005925-focal adhesion | 5 | 0.0312 | 1.506 | *JUP, HSPA5, IQGAP1, CALR, HSP90B1* | 0.179 |
| **Cellular Component** | GO:0005694 : chromosome | 4 | 0.004 | 2.395 | *CDCA5, NUSAP1, HMGB2, MCM4* | 0.343 |
| **Cellular Component** | GO:0072686 : mitotic spindle | 3 | 0.006 | 2.243 | *NUSAP1, CDC7, PKD2* | 0.409 |
| **Cellular Component** | GO:0034663-endoplasmic reticulum chaperone complex | 2 | 0.0300 | 1.523 | *HSPA5, HSP90B1* | 0.179 |
| **Molecular Function** | GO:0005515-protein binding | 52 | 0.0090 | 2.046 | *MS4A3, CHD7, HMGB2, F13A1, PKD2, MPO, DSTYK, NUSAP1, CTSG, AP3S1, PDE8A, SRGN, PRKCI, EGFL7, HGF, TMEM170B, RNASE2, AP5B1, CDC25A, LAT2, DNAJC3,*  *PTP4A3, DDAH2, FSCN1, TRIM58, MCM4, TLR4, SLC25A13, LY6E, FKBP5, FTO, CFH, TROAP, CDCA5, LIN7A, IQGAP1, PPM1F, PLD3, HSP90B1, BRD7, ELANE,*  *LNPK, BPTF, JUP, HSPA5, ATAD2, CDC7, UEVLD, PFKL, CALR, RAD18, SIPA1* | 0.237 |
| **Molecular Function** | GO:0016787-hydrolase activity | 17 | 0.0023 | 2.638 | *HSPA5, HGF, ATAD2, CHD7, RNASE2, ABCA13, CDC25A, PPM1F, PLD3, HSP90B1, PTP4A3, DDAH2, MCM4, CTSG, TLR4, PDE8A, ELANE* | 0.211 |
| **Molecular Function** | GO:0019899-enzyme binding | 14 | 0.0084 | 2.074 | *HSPA5, JUP, IQGAP1, PKD2, CDC25A, HSP90B1, DNAJC3, PFKL, TRIM58, CTSG, CALR, PDE8A, ELANE, RAD18* | 0.237 |
| **Molecular Function** | GO:0005524-ATP binding | 10 | 0.0340 | 1.469 | *PRKCI, DSTYK, PFKL, HSPA5, CHD7, ATAD2, MCM4, CDC7, ABCA13, HSP90B1* | 0.502 |
| **Molecular Function** | GO:0051087-protein-folding chaperone binding | 4 | 0.0080 | 2.098 | *DNAJC3, HSPA5, CALR, CDC25A* | 0.237 |
| **Molecular Function** | GO:0061775-cohesin loader activity | 4 | 0.0103 | 1.985 | *HSPA5, CHD7, ATAD2, MCM4* | 0.237 |
| **Molecular Function** | GO:0019207-kinase regulator activity | 4 | 0.0598 | 1.224 | *DNAJC3, HSPA5, IQGAP1, PDE8A* | 0.640 |
| **Molecular Function** | GO:0044183-protein folding chaperone | 3 | 0.0229 | 1.640 | *HSPA5, CALR, HSP90B1* | 0.378 |
| **Molecular Function** | GO:0051082-unfolded protein binding | 3 | 0.0547 | 1.262 | *HSPA5, CALR, HSP90B1* | 0.611 |
| **Molecular Function** | GO:0000510-H3-H4 histone complex chaperone activity | 4 | 0.0098 | 2.008 | *HSPA5, CHD7, ATAD2, MCM4* | 0.237 |

**Supplementary Table 8:** Enriched Reactome pathways (terms) among the 60 DEGs. Columns include: the Reactome identifier and pathway name (Term), number and percentage of DEGs involved (Count, %), unadjusted p-value (PValue), the list of contributing genes (Genes), and the adjusted p-value using False Discovery Rate (FDR).

| **Terms:** | **Count** | **%** | **P-Value** | **Genes** | **FDR** |
| --- | --- | --- | --- | --- | --- |
| R-HSA-381119 Unfolded Protein Response (UPR) | 4 | 6.70 | 0.0050 | *DNAJC3, HSPA5, CALR, HSP90B1* | 0.249 |
| R-HSA-6785807 Interleukin-4 and Interleukin-13 signaling | 4 | 6.70 | 0.0072 | *HGF, FSCN1, F13A1, HSP90B1* | 0.229 |
| R-HSA-176187 Activation of ATR in response to replication stress | 3 | 5.00 | 0.0081 | *MCM4, CDC7, CDC25A* | 0.209 |
| R-HSA-69206 G1/S Transition | 4 | 6.70 | 0.0098 | *DHFR, MCM4, CDC7, CDC25A* | 0.219 |
| R-HSA-453279 Mitotic G1 phase and G1/S transition | 4 | 6.70 | 0.0143 | *DHFR, MCM4, CDC7, CDC25A* | 0.169 |

**Supplementary Table 9:** **Drug sensitivity analysis reveals differential therapeutic vulnerabilities using OncoPredict.**

| **IT-8** | **Drug*** | **P-value** | **Drug Targets** | **Targets Pathway** |
| --- | --- | --- | --- | --- |
|  | **MK-1775_1179** | P< 0.05 | *WEE1, PLK1* | Cell cycle |
|  | **MK-8776_2046** | P< 0.05 | *CHEK1, CHEK2, CDK2* | Cell cycle |
|  | **VE821_2111** | P< 0.05 | *ATR* | Genome integrity |
|  | **Luminespib_1559** | P< 0.05 | *HSP90* | Protein stability and degradation |
|  | **Afuresertib_1912** | P< 0.05 | *AKT1, AKT2, AKT3* | PI3K/MTOR signaling |
|  | **Uprosertib_1553** | P< 0.05 | *AKT1, AKT2, AKT3* | PI3K/MTOR signaling |
|  | **Nutlin-3a_1047** | P< 0.05 | *MDM2* | p53 pathway |
| **IT-21** | **Drug*** | **P-value** | **Drug Targets** | **Targets Pathway** |
|  | **AZD2014_1441** | P< 0.05 | *mTORC1, mTORC2* | PI3K/MTOR signaling |
|  | **AZD8055_1059** | P< 0.05 | *MTORC1, MTORC2* | PI3K/MTOR signaling |
|  | **Afuresertib_1912** | P< 0.05 | *AKT1, AKT2, AKT3* | PI3K/MTOR signaling |
|  | **Dactolisib_1057** | P< 0.05 | *PI3K (class 1), MTORC1, MTORC2* | PI3K/MTOR signaling |
|  | **Rapamycin_1084** | P< 0.05 | *MTORC1* | PI3K/MTOR signaling |
|  | **Tozasertib_1096** | P< 0.05 | *AURKA, AURKB, AURKC, others* | Mitosis |
|  | **Sabutoclax_1849** | P< 0.05 | *BCL2, BCL-XL, BFL1, MCL1* | Apoptosis regulation |
| **IT-13+22** | **Drug*** | **P-value** | **Drug Targets** | **Targets Pathway** |
|  | **Dabrafenib_1373** | P< 0.05 | *BRAF* | ERK MAPK signaling |
|  | **Sapitinib_1549** | P< 0.05 | *EGFR, ERBB2, ERBB3* | EGFR signaling |

* Database: https://www.cancerrxgene.org/

**Supplemental Figure S1**

**
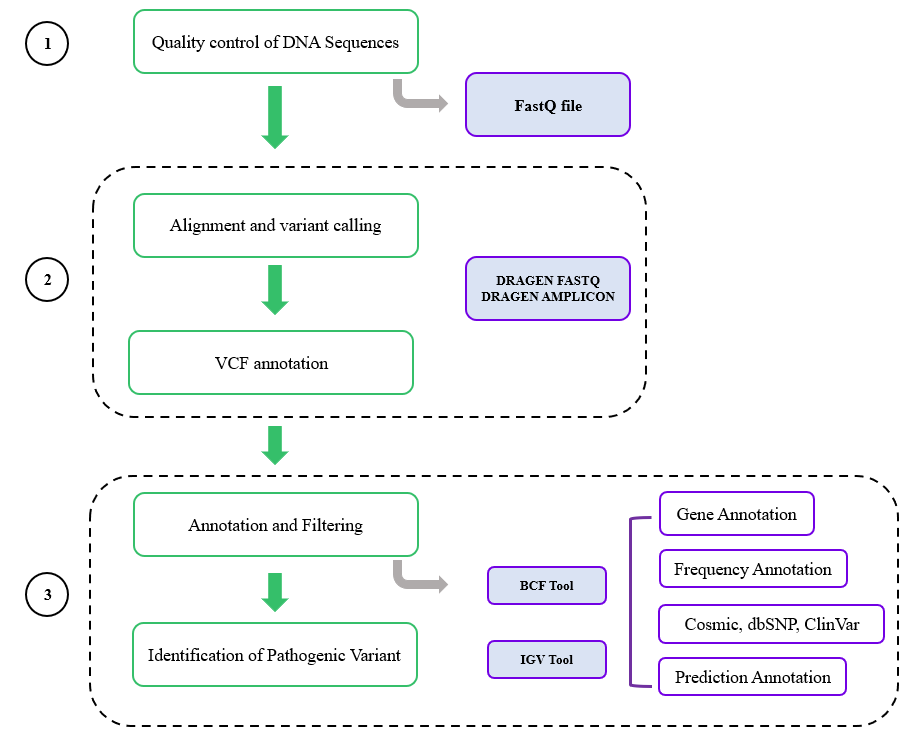
**

**Supplemental Figure S1:** Main stages of analysis and processing of genomic data generated with NGS: (1) Quality control of raw data, (2) sequence alignment, variant calling, and annotation in VCF files, (3) annotation and filtering of genetic variants using BCF tools + (Processing and filtering in R), and graphical visualization with IGV for variant identification and classification.

**Supplemental Figure S2**


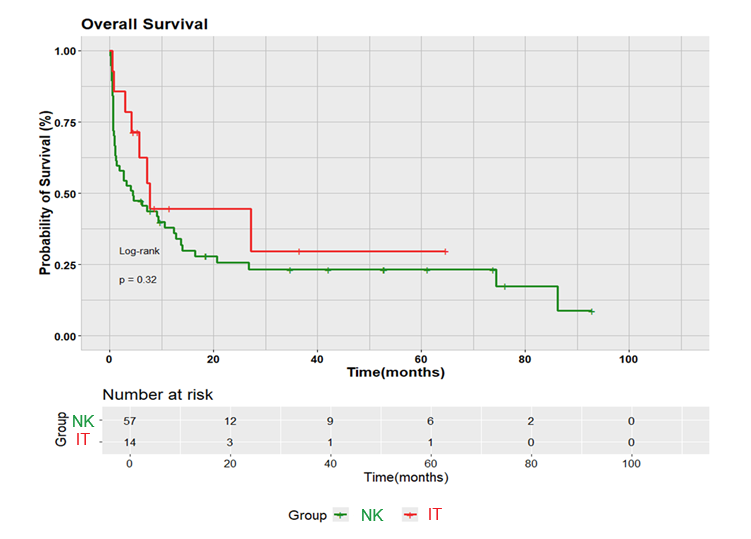


**Supplemental Figure S2:** Overall survival analysis between the NK and IT groups of adult patients with acute myeloid leukemia treated with intensive therapy. OS analysis of 71 patients in our cohort, including 57 in the NK group and 14 in the IT group. The log-rank test was used to differentiate the groups.
